# Subtype and prognostic analysis of immunogenic cell death-related gene signature in prostate cancer

**DOI:** 10.3389/fonc.2023.1160972

**Published:** 2023-06-06

**Authors:** Zhen Kang, Jiang-Bo Sun, Fei Lin, Xu-Yun Huang, Qi Huang, Dong-Ning Chen, Qing-Shui Zheng, Xue-Yi Xue, Ning Xu, Yong Wei

**Affiliations:** ^1^Department of Urology, Urology Research Institute, The First Affiliated Hospital, Fujian Medical University, Fuzhou, China; ^2^Department of Urology, National Region Medical Centre, The First Affiliated Hospital, Fujian Medical University, Fuzhou, China; ^3^Fujian Key Laboratory of Precision Medicine for Cancer, The First Affiliated Hospital, Fujian Medical University, Fuzhou, China

**Keywords:** immunogenic cell death, prostate cancer, immune infiltration, cancer subtype, prognostic signature

## Abstract

**Background:**

Immunogenic cell death (ICD) plays a vital role in tumor progression and immune response. However, the integrative role of ICD-related genes and subtypes in the tumor microenvironment (TME) in prostate cancer (PCa) remains unknown.

**Materials and methods:**

The sample data were obtained from The Cancer Genome Atlas (TCGA), Gene Expression Omnibus (GEO), and Memorial Sloan Kettering Cancer Center (MSKCC) prostate cancer-related databases. We first divided the subtypes based on ICD genes from 901 PCa patients and then identified the prognosis- related genes (PRGs) between different ICD subtypes. Subsequently, all the patients were randomly split into the training and test groups. We developed a risk signature in the training set by least absolute shrinkage and selection operator (LASSO)–Cox regression. Following this, we verified this prognostic signature in both the training test and external test sets. The relationships between the different subgroups and clinical pathological characteristics, immune infiltration characteristics, and mutation status of the TME were examined. Finally, the artificial neural network (ANN) and fundamental experiment study were constructed to verify the accuracy of the prognostic signature.

**Results:**

We identified two ICD clusters with immunological features and three gene clusters composed of PRGs. Additionally, we demonstrated that the risk signature can be used to evaluate tumor immune cell infiltration, prognostic status, and an immune checkpoint inhibitor. The low-risk group, which has a high overlap with group C of the gene cluster, is characterized by high ICD levels, immunocompetence, and favorable survival probability. Furthermore, the tumor progression genes selected by the ANN also exhibit potential associations with risk signature genes.

**Conclusion:**

This study identified individuals with high ICD levels in prostate cancer who may have more abundant immune infiltration and revealed the potential effects of risk signature on the TME, immune checkpoint inhibitor, and prognosis of PCa.

## 1 Introduction

Prostate cancer (PCa) ranks as the second most common male cancer worldwide, with approximately 1.3 million new cases diagnosed each year (1). As PCa is an androgen-dependent tumor in men, the combination of androgens and androgen receptors can promote tumor development. Consequently, androgen deprivation therapy (ADT) has become the primary drug therapy for PCa, effectively improving the 5-year survival rate of patients to nearly 100% (2). However, when a patient experiences tumor metastasis or uncontrolled prostate-specific antigen (PSA) levels, the tumor may develop hormone resistance, resulting in resistance to ADT agents and a decline in the 5-year survival rate to approximately 30%. Therefore, exploring new treatment approaches for advanced prostate cancer is crucial to address unmet clinical needs (3).

The progression, metastasis, and deterioration of tumors are closely related to immune responses. The immune system can eliminate tumors through various mechanisms, exerting a certain level of control. In recent years, immunotherapy has made significant advancements. Immune checkpoint inhibitors (ICPIs) have transformed the treatment landscape for most malignant tumors, but their positive therapeutic effects have yet to be identified in advanced prostate cancer (4).

In prostate cancer, sipuleucel-T is currently the only cancer vaccine approved by the US Food and Drug Administration for castration-resistant prostate cancer (CRPC). The IMPACT study results demonstrated that sipuleucel-T increased the overall survival of patients by 4.1 months (5). However, many immunotherapy trials have reported unsatisfactory results. In a phase I clinical trial of the CTLA-4 antibody ipilimumab, only 2 out of 14 advanced PCa patients experienced a PSA decline of ≥50% (6). Another phase II trial, CheckMate 650, investigated the combination of ipilimumab and nivolumab (7), which yielded a mere 25% objective response rate. In a phase III trial, the 1-year survival rate for PCa patients treated with ipilimumab was 46.5%, compared to 40.8% in the placebo group (8), a difference that was not statistically significant. These results suggest that the outcomes of immunotherapy in the treatment of PCa are inconsistent and deserve further exploration.

One reason for this phenomenon is that most prostate cancers are “cold” tumors with low T- cell infiltration (9), leading to an insufficient number of immune cells in the tumor immune microenvironment and, consequently, an unstable therapeutic effect of ICPIs. Some researchers have proposed developing new biomarkers based on the existing multiple immune activation pathways and stratifying the population according to immune response (10–12). This approach aims to explore the immunological characteristics of different population levels, ultimately achieving personalized immunotherapy in prostate cancer.

Immunogenic cell death (ICD) is a form of regulated cell death that can mediate the activation of innate and adaptive immune responses by coordinating a complex information exchange between dead cancer cells (DCCs) and immune cells (13). However, it remains unclear whether different ICD levels exist in prostate cancer. In theory, dead tumor cells are typically immune-tolerant or non-immunogenic, and under ICD induction, DCCs recruit antigen-presenting cells (APCs) and effector CD4+ and CD8+ T cells by releasing tumor-associated antigens and cytokines through damage-associated molecular patterns (DAMPs). ICD facilitates a transition in the tumor microenvironment from a non-inflammatory “cold” state to an inflammatory “hot” state (14), enhancing T- cell activation, which ultimately results in more effective immune-mediated tumor cell killing.

To thoroughly evaluate the association between prostate cancer and ICD response, we identified subgroups with different ICD levels in the prostate cancer population based on ICD-related genes. We used the differentially expressed genes (DEGs) between ICD groups to establish prognostic risk models to analyze the differences in prognosis and immune microenvironment among patients with various ICD levels. The results indicate that this new ICD-related risk model can be employed to predict the prognosis of prostate cancer and evaluate the immune environment.

## 2 Materials and methods

### 2.1 Data acquisition

We downloaded the fragments per kilobase per million (FPKM) data, clinical survival data, and tumor gene mutation-related data for 554 prostate cancer patients from The Cancer Genome Atlas (TCGA) database (https://portal.gdc.cancer.gov/). In addition, we downloaded the “GSE70770”, “GSE46602”, and “MSKCC” datasets from the Gene Expression Omnibus (GEO) database (https://www.ncbi.nlm.nih.gov/geo/) and The Fudan Data Portal for Cancer Genomics (https://data.3steps.cn/cdataportal/), which included a total of 454 second-generation sequencing data and matched clinical data for prostate cancer. All the specimens in the database were collected after radical prostatectomy. We accessed the Molecular Signatures Database (MSigDB) through the msigdb package in R and downloaded the “c2.cp.kegg.v7.4.symbols. gmt” file. More importantly, 33 human ICD-related genes were derived from the documentation of Garg AD et al. (15).

### 2.2 Processing of genetic data

After converting TCGA-PRAD FPKM data to TPM format, we used the “sva” package to merge the gene expression data of GSE70770, Memorial Sloan Kettering Cancer Center (MSKCC), and TCGA-PRAD, which was named “co-matrix”; the horizontal axis represents the patient ID of 901 cases, and the vertical axis represents gene names.

### 2.3 Processing of clinical data

Our clinical data were obtained from the TCGA-PRAD database, which was updated in April 2022, where “biochemical_recurrence” was used as the disease progression status and “days_to_first_biochemical_recurrence” was used as the disease progression time. For patients without recorded “days_to_first_biochemical_recurrence”, “days_to_last_followup” was used as the disease progression time and “has_new_tumor_events_information” as the disease progression status.

In GSE70770, “biochemical relapse” was used as the disease progression status, and “time to bcr” was used as the disease progression time. In GSE46602, “bcr” was used as the disease progression status and “bcr_free_time” was used as the disease progression time. In the MSKCC cohort, “Disease Free Status” was used as the indicator of disease progression and “Disease Free” was used as the time of tumor occurrence and progression.

### 2.4 ICD genes in PCa

The “maftools” package in R was utilized to process TCGA mutant gene data to obtain the mutation status of ICD gene in TCGA-PRAD. In addition, the copy number variation (CNV) profile in PCa was analyzed in “Perl” to delineate the ICD gene CNV situation in TCGA-PRAD. Finally, the “limma” package was used to analyze the ICD gene expression difference between normal and tumor tissues.

### 2.5 The first consensus clustering

The “ConsensusClusterPlus” package (16) in R was used to perform consensus clustering on the “co-matrix” described in Section 2.2, with clustering based on ICD genes. The main parameter settings were as follows: clusterAlg=pam; Short=spearman; seed=123456. In this study, the optimal number of clusters was evaluated between k = 2 and k = 9, and the process was replicated 1,000 times to ensure reliable results.

### 2.6 Compare different ICD clusters

The “survival” package was used to compare the clinical progression-free survival (PFS) of different ICD clusters, while gene set variation analysis (GSVA) was used to identify functional differences in pathways between the two clusters. The “limma” package was used to screen for ICD-related differentially expressed genes (ICD-DEGs) between the two ICD clusters, using a significance threshold of adjusted *p*< 0.05 and a log2 fold-change< 0.585. Kyoto Encyclopedia of Genes and Genomes (KEGG) pathway analysis was also performed on the identified ICD-DEGs. The single-sample gene set enrichment analysis (ssGSEA) approach was used to compare the infiltration composition of 23 immune cells in each TCGA-PRAD sample. Univariate Cox regression analysis was performed to identify prognosis- related ICD-DEGs (PRGs), and the PRGs were further analyzed to determine their potential as prognostic biomarkers for PCa.

### 2.7 The second consensus clustering

1) After extraction of the PRG expression profiles from the co-matrix, consensus clustering was performed using the “ConsensusClusterPlus” package in R, with the adjusted number of clusters set to 2–10. The key parameter settings were “clusterAlg=km; distance=euclidean, seed=123456”, and 1,000 iterations were performed. The most appropriate clustering number was determined based on the silhouette coefficient and discrimination between different subtypes. To distinguish the ICD cluster in “2.5”, it is referred to here as a gene cluster. 2) The “survival” package was used to investigate patient PFS between different gene clusters, and the “heatmap” package was utilized to compare clinical characteristics. 3) After clinical survival data and PRGs of all patients were combined, they were randomly divided into the training group and the testing group at a ratio of 7:3. In the training group, least absolute shrinkage and selection operator (LASSO) regression analysis was employed to minimize overfitting and to choose relevant variables among the PRGs. Multivariate Cox proportional hazards regression analysis was conducted to identify a risk signature (RS). The formula was as follows: riskScore = $\sum_{i=1}^{n}\beta_{\text{i}}*RG_{\text{i}}$ , where β_i_ represents the expression of the risk gene (RG) and *RG*_i_ represents the gene expression coefficient calculated from multivariate Cox regression. With the use of the median risk score as the cutoff, patients were divided into high- and low-risk groups in the training group, and the PFS of the two groups was compared to verify the accuracy of the RS. The RS accuracy was also confirmed in the testing group and external test set GSE46602. The “ROC” package was used for risk score analysis and receiver operating characteristic curve analysis. 4) Considering age, stage, Gleason score (GS), and risk score as survival-related factors, a nomogram was assembled based on these factors from samples in all patients. This nomogram provides clinicians with a quantitative approach to predict the survival of PCa patients. The calibration curve was drawn to verify the accuracy of the nomogram. 5) The “cor.test” function of the “psych” package was used to test the statistical significance of the correlation coefficient between ICD gene and RG in the co-matrix. 6) The pathological picture of the risk gene was obtained from the Human Protein Atlas (HPA) (https://www.proteinatlas.org/).

### 2.8 Risk signature and immune effect

1) The tumor immune microenvironment variations in low- and high-risk subtypes were analyzed by computing the immune score and tumor purity of each PCa sample using transcriptome data and the “estimate” package. 2) Based on the risk score calculated by RS, “reshape” and “corrplot” packages were used to evaluate the correlation between the risk score and immune checkpoint molecules and tumor mutational burden (TMB).

### 2.9 Artificial neural network

The mRNA expression matrix and platform sequencing data, recorded with hormone-sensitive prostate cancer (HSPC) and CRPC, were downloaded from the GEO database. A simple random sampling method without repetition was used to divide the dataset into two groups: the training set and the testing set, and note that this should be distinguished from the concepts in Section 2.7. The “limma” package was used to analyze the differential genes of CRPC and HSPC in the training set. Then, multiple decision trees of the random forest were trained on the PCa samples using the “randomForest” package. The different genes in the training set were processed to perform the random forest operation, with the parameters set as “seed=123456, ntree=500, MeanDecreaseGini=2”. Based on the selected random forest-related genes (RFGs), an artificial neural network (ANN) prediction model was established using the “neuralnet” package, and the receiver operating characteristic (ROC) curve was drawn using the “pROC” package. The prediction accuracy of the ANN model was analyzed and demonstrated in both the test set and the training set. Lastly, the “psych” package was used to test whether the correlation coefficient between RFGs and RG in co-matrix was statistically significant.

### 2.10 RNA extraction and qRT-PCR

RNA was extracted from RWPE-1/PC-3/C4-2/LNCaP cells using a TRIzol kit (Invitrogen, Carlsbad, CA, USA) according to the manufacturer’s specifications. After detection of the RNA concentration using a nanospectrophotometer, the extracted RNA was converted into cDNA using a reverse transcription kit (TransGen Biotech, Beijing, China). Specific primers for the target gene were designed in NCBI (**Supplementary Table 1**) and synthesized by Shangya Biotechnology Co., Ltd. (Fuzhou, China). Appropriate amounts of cDNA, gene-specific primers, ddH_2_O, and 2× Taq Pro Universal SYBR qPCR Master Mix were mixed and transferred to PCR tubes. All qPCRs were set up in quadruplicate, with each tube containing a 20-μl reaction system. The reaction conditions were as follows: 95°C for 30 s, 95°C for 5 s, and 60°C for 30 s, for a total of 40 cycles. To calculate the gene expression levels, the following steps were taken: 1) the Ct mean for each gene of each sample was calculated. 2) The Ct value of the target gene in each sample was subtracted from the Ct value of the internal reference molecule (GAPDH) in the same sample to obtain the ΔCt value of the target molecule for each sample in each group. 3) The ΔCt arithmetic mean of the target gene in all samples was calculated separately. 4) The ΔCt value of the target molecule in each sample of each group (Step 2) was subtracted from the mean ΔCt value of the target molecule in all samples (Step 3) to obtain the ΔΔCt value of the target gene in each sample of each group. 5) After the calculation of 2^−ΔΔCt^, the relative expression of the target gene in each sample of each group was finally obtained. In RWPE-1 and C4-2, radioimmunoprecipitation assay (RIPA) buffer and the addition of appropriate protease inhibitors were used to extract total proteins from each cell type. After the protein concentration was measured using a bicinchoninic acid (BCA) protein concentration assay kit, the normal electrophoresis, membrane transfer, blocking, and the corresponding protein concentration were determined. The protein level was detected by incubating the antibody, which was purchased from ImmunoWay Biotechnology Company (Plano, TX, USA).

### 2.11 The tools

R version 4.1.1 was used for analysis in this study and the complete raw data and relevant code are provided in the **Supplementary Data 1**. A significance level of *p*< 0.05 was used to determine statistical significance (* *p*< 0.05; ** *p*< 0.01; *** *p*< 0.001). The procedure of this study is summarized in **Table 1**.

**Table 1:**
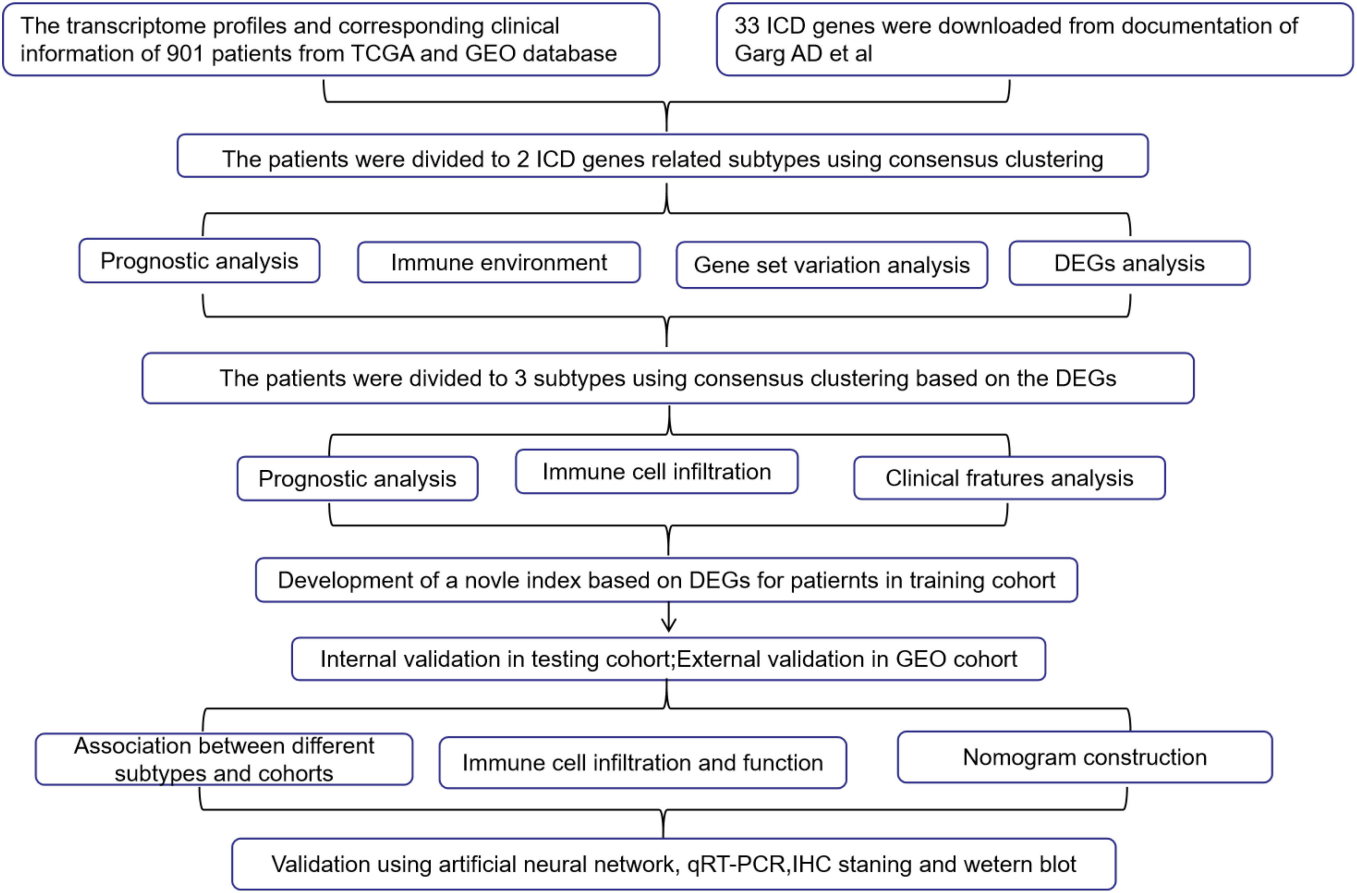


## 3 Results

### 3.1 ICD and PCa

**Figure 1:**
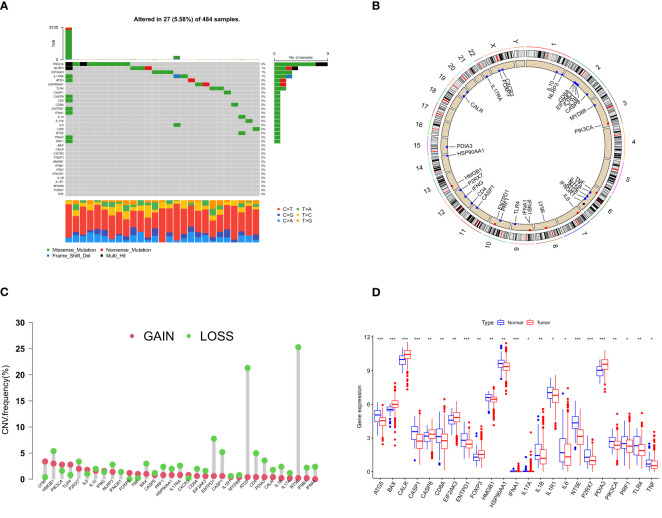
ICD- related gene mutation and CNV analysis in PCa. **(A)** Mutation frequency of 33 ICD genes in TCGA-PRAD samples. The number on the right indicates the mutation frequency of each gene. The bar chart on the right shows the proportion of mutations. The stacked bar chart on the bottom shows the fraction of conversions. **(B)** The frequency of CNV of ICD genes in human chromosomes. **(C)** CNV gain and loss pattern of ICD gene. Green circles on the top represent more “loss” than “gain” in the CNV of the target gene and vice versa. **(D)** Differentially expressed ICD genes in normal and tumor tissues. The lines in the boxes represent median value, and black dots show outliers. The asterisks represent the statistical *p*-value (*p< 0.05; **p< 0.01; ***p< 0.001). ICD, immunogenic cell death; CNV, copy number variation; PCa, prostate cancer.

We identified 33 human ICD-related genes through a comprehensive literature review, all of which can function as ICD-related danger signals or regulatory molecules. In TCGA-PRAD samples, 19 ICD genes exhibited varying degrees of mutation, with the majority being missense mutations, and an overall mutation frequency of 5.57% (**Figure 1A**). Among these, PIK3CA had the highest mutation frequency. Concurrently, we observed different degrees of DNA copy number variation in these ICD genes (**Figure 1B**). HMGB1, HSP90, IL17RA, CD8A, CALR, IL1B, IL17A, IFNB1, etc. displayed extensive CNV deletions, while 32 genes also demonstrated CNV on chromosomes (**Figure 1C**). Moreover, 23 ICD genes exhibited significant differential expression between benign tumors (n = 52) and prostate cancer (n = 501) (**Figure 1D**). These results may suggest a potential association between ICD and prostate cancer.

### 3.2 ICD subtypes in PCa

**Figure 2:**
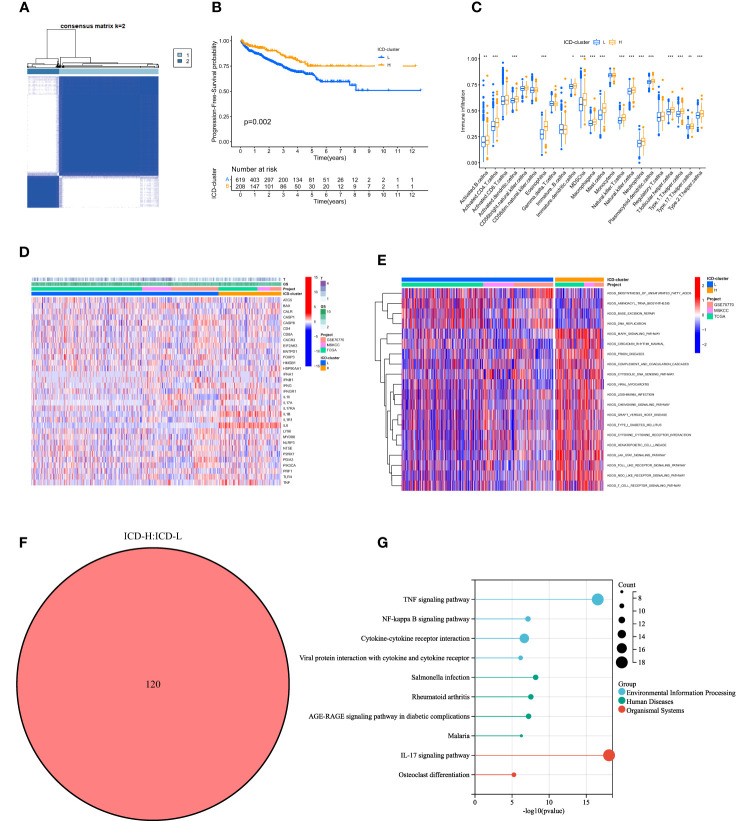
Comparison of the clinical traits, survival status, and tumor immune microenvironment between different cluster subtypes obtained by consensus clustering. **(A)** Consensus matrix heatmap defining two clusters (k = 2) and their correlation area. **(B)** Kaplan–Meier curve shows that there were significant survival differences between ICD-L and ICD-H (*p* = 0.002). **(C)** The abundance of each TME infiltrating cell in two clusters. The upper and lower ends of the boxes represent the interquartile range of values. The lines in the boxes represent the median value, and black dots show outliers. The asterisks represent the statistical *p*- value (*p< 0.05; **p< 0.01; ***p< 0.001). **(D)** Differences in clinicopathologic features and expression levels of ICD genes between the two distinct subtypes. **(E)** GSVA of biological pathways between two distinct subtypes, in which red and blue represent activated and inhibited pathways, respectively. **(F)** 120 DEGs between ICD-L and ICD-H. **(G)** KEGG pathways of differentially expressed genes. ICD, immunogenic cell death; TME, tumor microenvironment; GSVA, gene set variation analysis; DEGs, differentially expressed genes; KEGG, Kyoto Encyclopedia of Genes and Genomes.

The co-matrix incorporated nearly 18,000 gene expression data from 901 patients, from which we extracted 33 ICD genes to generate an ICD gene expression profile (**Supplementary Table 2**). In this dataset, consensus clustering divided the 901 patients into two subgroups (**Supplementary Table 3**), designated as ICD-H and ICD-L (**Figure 2A**). Kaplan–Meier (K-M) analysis results demonstrated a significant difference in PFS between the ICD-H and ICD-L groups (*p* = 0.002) (**Figure 2B**). The immune tumor microenvironment (TME) of ICD-H exhibited a more abundant infiltration of T cells, B cells, and dendritic cells (**Figure 2C**). To explain the phenomenon of population stratification from an ICD- level perspective, we analyzed the distribution of clinical information and ICD genes in different populations (**Figure 2D**). We identified general differences between the ICD-H and ICD-L subtypes, such as higher expression of IL6, IL1B, IL10, and other interleukins in ICD-H. In contrast, HMGB1 was more active in ICD-L. GSVA revealed that the main functional pathways in ICD-H included the T- cell receptor signaling pathway, MAPK signaling pathway, and JAK–STAT signaling pathway, indirectly indicating an active immune function in ICD-H. Meanwhile, ICD-L was primarily involved in DNA synthesis, base excision repair, and unsaturated fatty acid synthesis (**Figure 2E**). For further comparative analysis, we screened 120 ICD-DEGs (*p*< 0.05, LogFC > 0.585) between ICD-H and ICD-L by combining whole-genome data from the 901 patients (**Figure 2F**). The functions of these DEGs were mainly concentrated in the TNF signaling pathway, NF-kappa B signaling pathway, IL17 signaling pathway, and other related pathways (**Figure 2G**).

### 3.3 Gene subtypes in PCa

**Figure 3:**
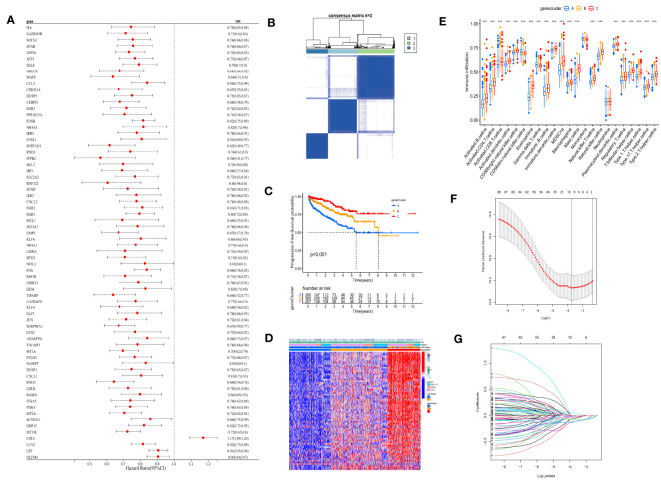
Consensus clustering and LASSO analysis were performed according to PRGs. **(A)** Univariate Cox regression screened 70 PRGs. **(B)** Consensus matrix heatmap defining three clusters (k = 3) and their correlation area. **(C)** Kaplan–Meier curve shows that there were significant survival differences between gene clusters A, B, and C (*p*< 0.001). **(D)** Differences in clinicopathologic features and expression levels of PRGs between the three gene clusters. **(E)** The abundance of each TME infiltrating cell in three gene clusters. **(F)** LASSO coefficient curves were selected with simulation parameters set to 1,000. **(G)** Tenfold cross-validation of selecting tuning parameter in the LASSO model, and there were four variables (risk gene) left. LASSO, least absolute shrinkage and selection operator; PRGs, prognosis-related genes; TME, tumor microenvironment. (*** p<0.001).

Univariate Cox regression analysis was performed on the 120 DEG dataset, yielding a total of 70 prognosis-related genes (PRGs). Based on PRGs (**Supplementary Table 4**), consensus clustering of the 901 patients was conducted, dividing the population into three groups (A, B, and C) (**Figure 3A**). K-M survival analysis demonstrated significant survival differences among the three groups (*p*< 0.001) (**Figure 3B**), with group C exhibiting the best survival prognosis. Differences in gene expression among the three groups were also observed in the heatmap, as well as a high overlap between group C and the ICD-H group (**Figure 3C**), indicating more abundant immune cell infiltration (**Figure 3D**). In this study, the cohort of patients with complete survival information (survival time, survival status, and PRG expression) in the co-matrix was divided into a 7:3 ratio, randomly assigning them to a training set (n = 579) (**Supplementary Table 5**) and a test set (n = 248) (**Supplementary Table 6**). With the use of the LASSO regression method in the training set, and setting a 10-fold cross-validation, the optimal model was obtained (**Figures 3E, F, G**).

### 3.4 Risk signature

**Figure 4:**
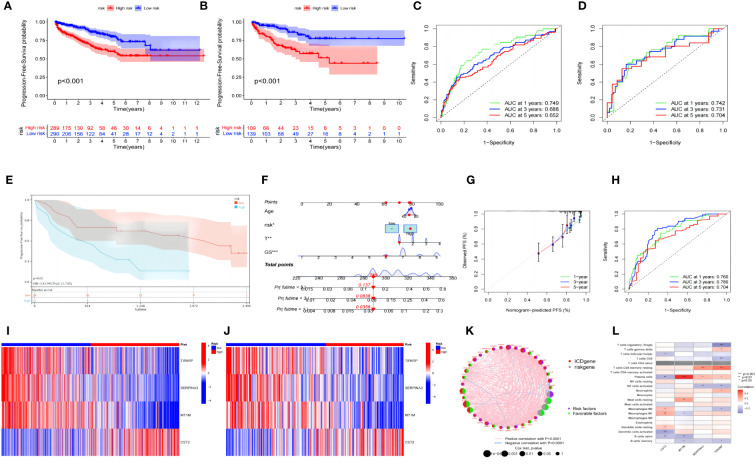
Prognosis value of the risk model in the training, test, and external test sets. **(A, B)** Kaplan–Meier survival curves of survival probability of patients between low- and high-risk groups in the training and test sets, respectively. **(C, D)** ROC curves to predict the sensitivity and specificity of 1-, 3-, and 5-year survival according to the risk score in the training and test sets, respectively. **(E)** Kaplan–Meier survival curves of survival probability of patients between low- and high-risk groups in the external test set. **(F)** Prognostic nomogram assembled from entire sets to predict 1-, 3-, and 5-year survival rates for PCa patients. **(G)** The calibration chart of the nomogram for entire set. **(H)** ROC curves of the prognostic nomogram for 1, 3, and 5 years in the entire set. **(I, J)** Distribution pattern of the expression levels of the four risk genes in the training and test sets. **(K)** Interaction between ICD genes and risk genes in PCa. The line connecting ICD genes and risk genes indicates their interaction, and the thickness of the line indicates the correlation strength between every gene. Purple and green represent negative and positive correlations, respectively. **(L)** Correlations between the abundance of immune cells and four risk genes in the risk signature model. ROC, receiver operating characteristic; PCa, prostate cancer; ICD, immunogenic cell death.

After screening the four risk genes (TIPARP, SERPINA3, MT1M, and CST2), each sample acquired a risk score according to the following formula: risk_score = exp(CST2) * 0.1095 − exp(TIPARP) * 0.2893 − exp(SERPINA3) * 0.2235 − exp(MT1M) * 0.1915. The median risk score (−3.9943) in the training set was set as the cutoff risk score to distinguish patients’ risk levels as high or low. Subsequently, survival analysis in the training and test groups suggested that the risk stratification determined by this signature could help predict PFS (**Figures 4A, B**). ROC curves were used to assess the sensitivity and specificity of the risk scores. The results were evaluated according to the area under the curve (AUC). The 1-, 3-, and 5-year AUC values of the training group were 0.749, 0.688, and 0.652, respectively (**Figure 4C**), while those of the test set were 0.742, 0.731, and 0.704, respectively (**Figure 4D**). As further validation of the risk signature, notably, the results in the external set (GSE46602) were consistent with those in the training group; patients with high risk had shorter PFS (**Figure 4E**).

The clinical data and risk scores of the entire data (training group and test group) were combined to construct a nomogram that could predict disease progression (**Figure 4F**). The ROC curve showed that the nomogram had good predictive ability for PFS, with high accuracy, and the AUC values of 1-, 3-, and 5-year survival were 0.760, 0.780, and 0.704, respectively (**Figure 4G**). The prediction ability of the nomogram for 1, 3, and 5 years was highly coincident with that of the ideal model (**Figure 4H**), and the C-index of the model was 0.73, indicating that the accuracy of the model was commendable. For the risk genes, TIPARP, SERPINA3, and MT1M were more actively expressed in the low-risk group in both the training set (**Figure 4I**) and the test set (**Figure 4J**), while CST2 was the only high-expression gene in the high-risk group. Combined with ICD gene analysis, it was found that CST2, as a high-risk factor, had a significant negative correlation with IL6, while TIPARP, SERPINA3, and MT1M had a significant positive correlation with IFNGR1, HSP90AA1, and HMGB1 (**Figure 4K**). The ICD level was strongly correlated with dendritic cell (DC) activity, and by combining analysis with immune cells, it was found that CST2 was significantly positively correlated with resting DC activity, while SERTAD3 was significantly positively correlated with activated DC activity. TIPARP and MT1M were also associated with macrophage and CD4+ T- cell activity (**Figure 4L**).

### 3.5 Association between different sets

**Figure 5:**
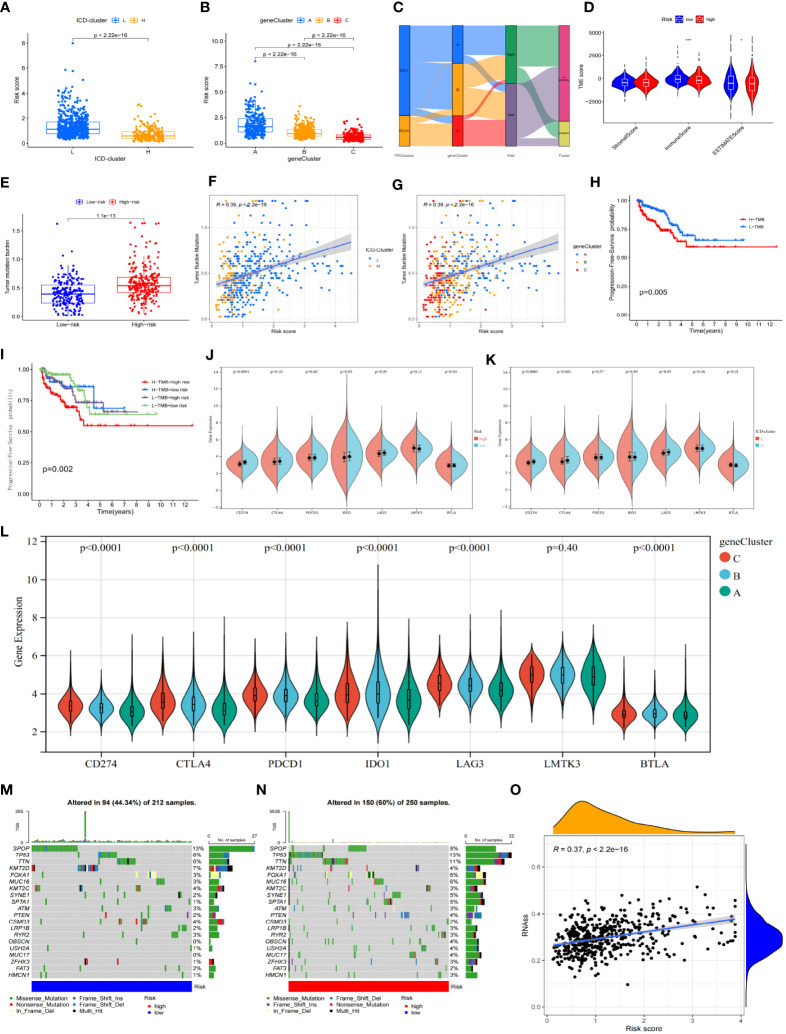
Associations and distinctions between every subgroup. **(A, B)** Comparison of risk scores between every group in the ICD cluster and gene cluster. **(C)** Alluvial diagram of every subtype distribution in groups with different risk groups and survival outcomes. **(D)** Correlations between risk score and both immune and stromal scores. **(E)** Comparison of TMB between high-risk and low-risk groups. **(F, G)** The association between risk score and TMB in the ICD cluster and gene cluster. **(H)** Kaplan–Meier survival analysis of the TMB in TCGA-PRAD cohort. **(I)** Kaplan–Meier survival analysis of four groups stratified by combining the TMB and the risk signature in the TCGA-PRAD cohort. **(J–L)** The association between ICPI and every subtype. The asterisks represent the statistical *p*-value (*p< 0.05; **p< 0.01; ***p< 0.001). **(M, N)** Waterfall plot of tumor somatic mutation established for those with high- and low-risk groups. **(O)** The association between risk score and RNAs. ICD, immunogenic cell death; TMB, tumor mutational burden; ICPI, immune checkpoint inhibitor.

The ICD-L group had a lower risk score than the ICD-H group (*p*< 0.001) (**Figure 5A**). There were also significant differences in risk scores between groups A, B, and C, with group C having the lowest risk score (*p*< 0.001) (**Figure 5B**). The “low-risk group C-ICD-H” population showed a high overlap (**Figure 5C**). Regarding the differences in the immune microenvironment between the high-risk and low-risk scores, the low-risk group had a higher immune cell infiltration score (**Figure 5D**) but a lower TMB (**Figure 5E**), as calculated by Estimate. The “ICD-H” (**Figure 5F**) and “group C” (**Figure 5G**) populations also had lower TMB levels, which corresponded to better survival prognosis (**Figure 5H**).

The combined analysis of risk scores and TMB found that patients in the “high-risk group-high TMB group” had the worst prognosis, with no significant difference in PFS between the other three combinations (**Figure 5I**). Higher expression of PD-L1 (CD274), IDO1, and LAG3 was observed in the low-risk group (**Figure 5J**) and also in the ICD-H group (**Figure 5K**), whereas higher expression of PD-1, PD-L1, CTLA4, IDO1, and LAG3 was observed in group C (**Figure 5L**). However, the high-risk population had a higher number of CNV levels (**Figures 5M, N**) and poorly differentiated stem cells (**Figure 5O**).

**Figure 6:**
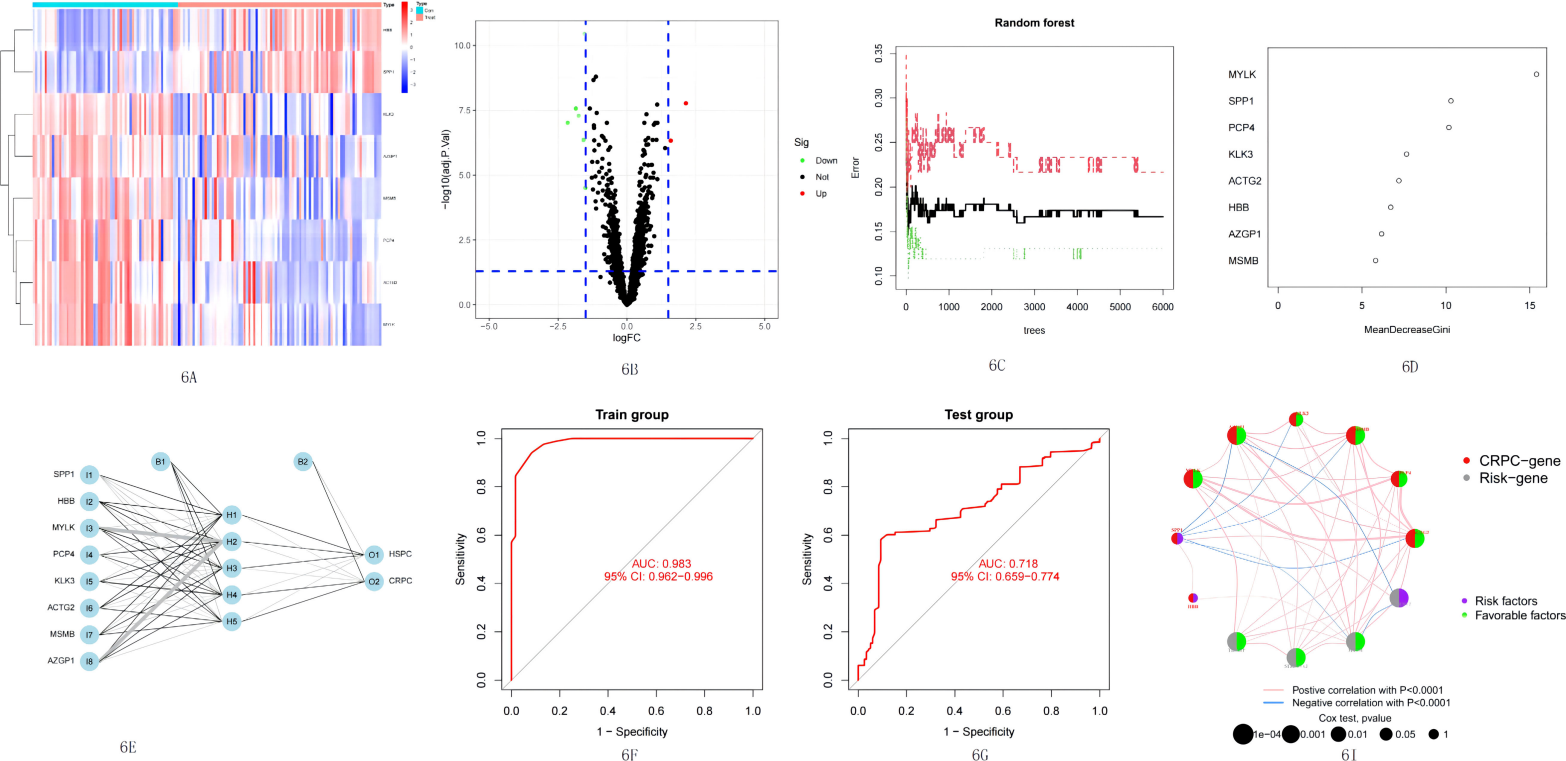
**(A)** Distribution pattern of the expression levels of the eight DEGs between CRPC and HSPC in the training set. **(B)** Volcanic map of differential genes between CRPC and HSPC. **(C)** Influence of the number of decision trees on the error rate. The x-axis is the number of decision trees and the y-axis is the error rate. **(D)** Ranking of input variables in the random forest model to classify CRPC and HSPC samples; RFGs are listed from the most important ones to the least ones based on MeanDecreaseAccuracy and MeanDecreaseGini. **(E)** Neural network topology of microarray training set* with eight input layers, five hidden layers, and two output layers. **(F, G)** In training set*, the ANN model achieved superior performance (AUC: 0.983). In test set*, the ANN model achieved an AUC of 0.718. The optimal threshold values are labeled at inflection points, and the sensitivities and specificities are in brackets. **(H)** Interaction between risk genes and CRPC genes in entire set. DEGs, differentially expressed genes; CRPC, castration-resistant prostate cancer; HSPC, hormone-sensitive prostate cancer; RFGs, random forest-related genes; ANN, artificial neural network; AUC, area under the curve.

In conclusion, there may be a group of prostate cancer patients with a good prognosis and high ICD levels, represented by group C, which can be identified by the risk prognosis model presented in this study. This group may potentially benefit from immunotherapy, as they display higher expression of immune checkpoint genes, lower TMB, and more abundant immune cell infiltration. Identifying these patients may help tailor personalized treatment approaches, ultimately improving outcomes for prostate cancer patients.

### 3.6 Verified with ANN

We aimed to improve the predictive power of prostate cancer progression by utilizing the GEO platform to acquire datasets. Due to the limited availability of complete survival data, we decided to combine datasets that recorded state changes to create a larger dataset for analysis. Nine datasets were obtained from the GEO platform. These datasets were randomly assigned to create five training sets (GSE2443, GSE5377, GSE5803, GSE29650, and GSE32269) with a total of 154 samples, which were later reduced to 144 samples after removing normal prostate and incomplete data samples. These samples included 60 HSPC samples and 84 CRPC samples. Four test sets (GSE74685, GSE6811, GSE46002, and GSE60329) were also created, containing 370 PCa samples, including 118 HSPC samples and 196 CRPC samples. The remaining 56 samples had no clear information recorded or belonged to normal prostate tissue.

Following the pre-set screening conditions (*p*< 0.05, log2 FC< 1.5), eight differentially expressed genes (MYLK, SPP1, ACTG2, PCaP4, MSMB, KLK3, HBB, and AZGP1) were identified based on the training group (**Supplementary Table 7**) (**Figures 6A, B**). SPP1 and HBB were upregulated genes in CRPC, while the others were downregulated genes. Random forest tree analysis was used to screen genes from DEGs that could effectively distinguish PCa types (HSPC/CRPC). The optimal number of decision trees was found to be 68 (**Figures 6C, D**). All eight DEGs were included in the RFG for subsequent model construction. The neural network algorithm was used to optimize the weight value of each RFG, and the final ANN model was obtained (**Figure 6F**). In the control group (HSPC), 53 of 60 samples were accurately predicted, while in the experimental group (CRPC), 81 of 84 samples were accurately predicted. The AUC of the ANN model was found to be 0.983 (**Figure 6F**).

The accuracy of the ANN model was tested in the test sets, and it was found that 83 out of 118 control samples were accurately predicted, and 123 out of 196 samples in the experimental group were accurately predicted, with an AUC of 0.718 (**Figure 6G**). These results confirmed that the eight selected genes were effective in predicting the progression of prostate cancer. Finally, the potential association between RFGs and risk scores (RS) was analyzed, finding that SPP1 and HBB, two CRPC upregulated genes, were significantly positively correlated with the risk gene CST2. TIPARP, SERPINA3, and MT1M, the three “protective” factors, were significantly positively correlated with CRPC downregulated genes (**Figure 6H**). This suggests a close association between the four prognostic model genes and the clinical progression of prostate cancer patients.

### 3.7 Experimental verification

**Figure 7:**
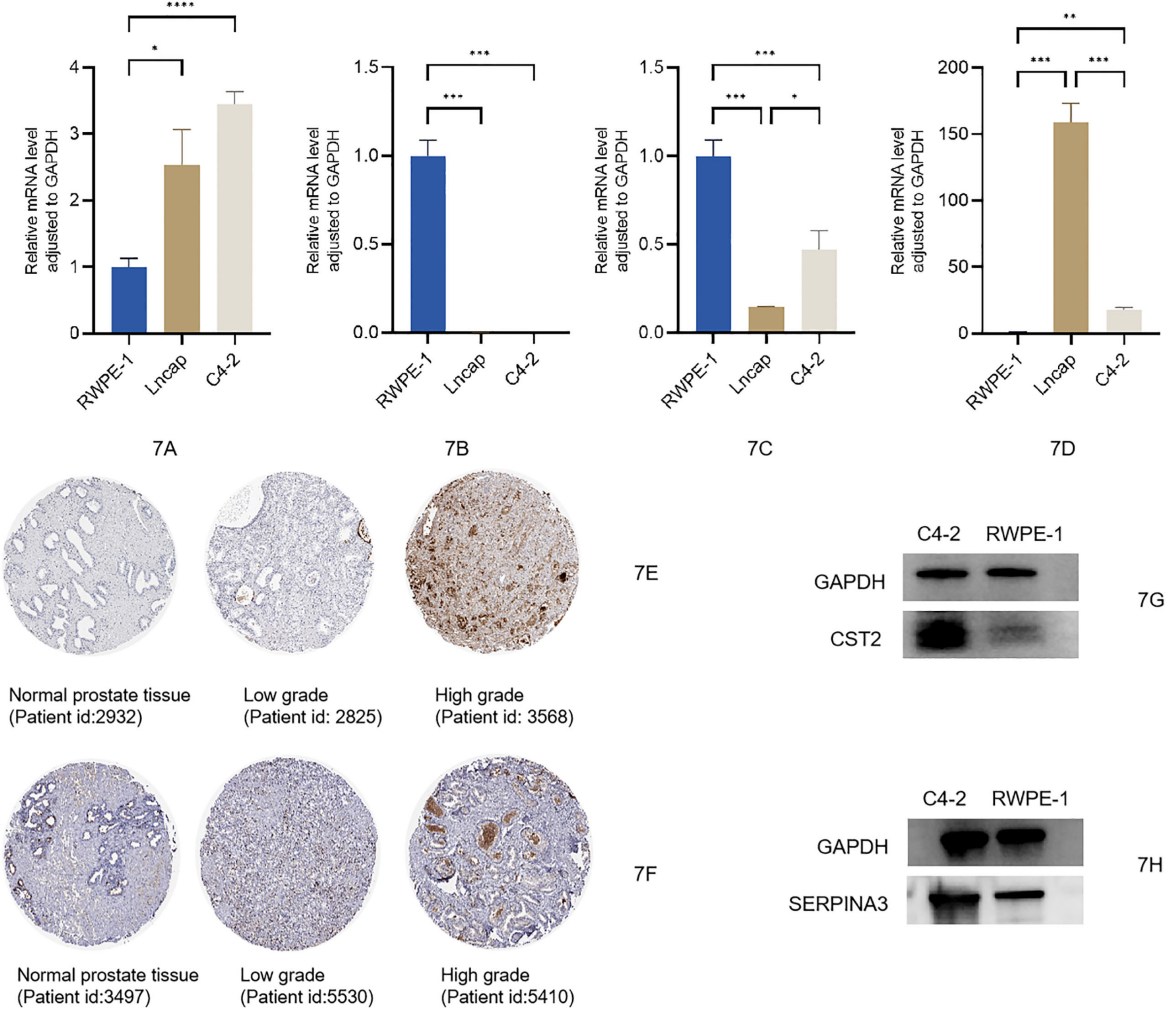
**(A–D)** Expression levels of mRNA between normal prostatic epithelial cells and PCa cells of CST2, MT1M, TIPARP, and SERPINA3. Protein expression levels of CST2 and SERPINA3 **(E–H)**. PCa, prostate cancer.

We used qRT-PCR to measure the relative mRNA expression levels of four risk genes (TIPARP, SERPINA3, MT1M, and CST2) in the normal prostate tissue cell RWPE-1 and in different tumor cell states such as LNCaP and C4-2 cell lines. As the results show, the mRNA expression levels of CST2 were increased as the cell status changed from normal to tumor. Levels of MT1M and TIPARP were prominently lower in LNCaP and C4-2 cells compared with RWPE-1. The SERPINA3 level was significantly increased in the LNCaP cell line but was lower in C4-2 and RWPE-1 cell lines (**Figures 7A–D**); the relevant raw data are available in **Supplementary Table 8**. Moreover, after searching the immunohistochemistry (IHC) staining results, the pictures for CST2 and SERPINA3 in normal and tumor tissues in the HPA database were similar. The relative protein expression levels of CST2 in the tissue of PCa were remarkably higher in comparison with those in the normal tissue (**Figure 7E**), and there were similar results for SERPINA3 in the corresponding tissues (**Figure 7F**). Western blotting (WB) results also showed that the expression of CST2 (**Figure 7G**) and SERPINA3 (**Figure 7H**) in the C4-2 cell line was significantly higher than that in prostate cell RWPE-1. The complete WB picture is displayed in the **Supplementary Data 2**.

## 4 Discussion

Currently, the standard of care (SOC) for hormone-sensitive prostate cancer mainly involves the combination of an androgen deprivation drug with endocrine drugs such as ADT combined with abiraterone or apalutamide. Other treatments such as ADT combined with paclitaxel or radiotherapy can also be used in conjunction with SOC. However, when the tumor develops bone metastasis or late-stage biochemical recurrence, i.e., progressing to the CRPC stage, the efficacy of SOC rapidly declines. Even with aggressive intervention, the median survival is only 2–3 years or even shorter. Although differentiated therapies such as PARP (poly ADP-ribose polymerase) inhibitors and immunosuppressants have been actively explored in CRPC, they have limitations in the application population and overall unstable effects. Therefore, the development of new biomarkers to identify patients who would benefit from ICPIs is a significant focus in prostate cancer research. Prostate cancer is often considered a “cold” tumor with relatively few somatic mutations and a lack of T- cell infiltration, which results in the unstable effect of ICPIs. This has been verified in numerous clinical trials. The “one-size-fits-all” treatment regimen for all patients is unlikely to be successful, and it is crucial to find new biomarkers to screen the ICPI benefit population. Current influencing factors for prostate cancer ICPI include TMB, microsatellite instability/mismatch repair (MSI/MMR), and cyclin-dependent kinase 12 gene, but more biomarkers affecting ICPI are still being studied and explored. So far, there has been no study on the ICD subtype. A recent study by Vinay Sagar et al. (17) found that EPHB4 can change the prostate TME by regulating the ICD response and improving the ICPI effect. This discovery inspired the researchers to combine the ICD gene found in previous studies and to explore the potential link between the development of prostate cancer and ICD. The integration of the ICD gene in the research could help identify new biomarkers and potential therapeutic targets for prostate cancer patients. This research may lead to a better understanding of the relationship between ICD and prostate cancer progression and may contribute to the development of more personalized treatment strategies for patients who would benefit from ICPI therapy.

This study found a significant difference in the rate of disease progression between two subgroups of patients classified according to ICD genes. The ICD-H group, which had a more active adaptive immune response, showed better survival benefits when compared to the ICD-L group. The DCs, T cells, and B cells were more abundant in this group, and the patients demonstrated more active cell functions in pathways essential for immune activities, such as T- cell receptors, NOD-like receptors, and Toll-like receptors. To better identify the ICD-H population, a prognostic risk model was developed to divide patients into high- and low-risk groups. High-risk patients had more rapid disease progression and a high overlap with the ICD-L group. Among the four critical signatures in the RS model, CST2 was the only gene that increased the risk of patient prognosis. CST2 is a cysteine protease inhibitor found in various human body fluids and secretions. Previous studies by Xiaohan Ren, Anqi Cheng, and others also identified CST2 as a risk factor for prostate cancer.

In this study, CST2 was found to be highly expressed in high-risk individuals in both the training and test groups. Combined with ICD genes, CST2 significantly negatively regulated the expression of IL6, which may indirectly inhibit T- cell proliferation and cytotoxic T-lymphocyte (CTL) activation. Regarding immune cells, CST2 was significantly negatively correlated with DC, T cells, and dendritic cells. To further explore the association between CST2 and prostate cancer development, machine learning was used to screen two CRPC highly expressed genes, SPP1 and HBB, which showed a significant positive correlation with CST2. Factors involved in the recognition of ICD-H subsets, affecting the activity of immune cells, and participating in the progression of CRPC are worthy of continued attention. Identifying and understanding these factors could lead to more effective and personalized treatment strategies for prostate cancer patients, potentially improving survival rates and overall patient outcomes.

According to the PRGs, patients were divided into three gene clusters (A, B, and C). Group C patients were similar to those in ICD-H, with lower risk scores and favorable survival prognoses. In ICD-L, however, two groups emerged (A and B), with group B patients having a better prognosis than group A. This finding indicates that population classification can be further improved. Group B may be a group of ICD “resistant” tumors between high and low levels of ICD, which can recruit immune cells but cannot successfully trigger an immune response, which is similar to the characteristics of immunosuppressive tumors. Tumors can be divided into four groups based on immune status and response to immunotherapy: hot tumors, immunomodulatory excluded tumors, immunomodulatory immunosuppressed tumors, and cold tumors. Different types may have the potential to transform into one another (18, 19). T-cell infiltration is abundant in hot tumors and immune-excluded tumors (20), but in the latter, T cells are sequestered in the tumor’s peripheral regions (21). We speculate that the ICD responses elicited by group B may be sequestered at the periphery of the tumor. This is reflected by the level of the TMB, PFS, and risk score in population B, which falls between groups A and C. It is possible that the immune responses generated by this population may not cause significant damage to tumor cells. The ideal ICD-induced response would involve more individuals in group B producing a rich immune microenvironment that works in conjunction with immunosuppressive therapy. As such, the differences between group B and group C warrant further investigation to better understand their distinct immunological responses and potential therapeutic implications.

The prognosis of the “high-risk + high TMB” group was the worst. As TMB increased in this research, patient risk scores also increased, while immune levels decreased. There was no ideal result in which high TMB would stimulate a high-level immune response. Some TMB is associated with a response to immunotherapy, and data show that high TMB tumors (e.g., melanoma and NSCLC) have an ICPI response rate of at least more than 15% (22). However, the TMB of all primary prostate cancers was low, which may be a factor for poor response to ICPI (23). Based on these results, we suggest that even in patients with high TMB, due to the scarcity of immune cells in prostate cancer itself, the “compensatory” immune response triggered by TMB cannot suppress the growth rate of the tumor itself. Therefore, only after changing the TME should we consider whether patients with high TMB prostate cancer can benefit from ICPI. By modulating four risk genes, especially CST2, it is possible to alter the level of tumor immunity, affecting the ICPI response rate.

PD-1/PD-L1 expression level is not considered equivalent to the ICI response level but is an important measure. The results of previous studies on the correlation between PD-1/PD-L1 expression levels and the efficacy of ICPI in prostate cancer are inconsistent. For example, in the phase II clinical trial KEYNOTE-199 (24), pembrolizumab monotherapy was compared with placebo treatment in 258 metastatic CRPC (mCRPC) patients, with response rates of only 5% and 3% in PD-L1-positive and PD-L1-negative cohorts, respectively. In the subsequent phase III clinical trial KEYNOTE-641 (25), pembrolizumab did not show improvement in PFS or OS compared to the placebo group. The phase II clinical trial CheckMate 650 investigated the combined treatment effect of ipilimumab and nivolumab, with the combination producing only a 25% objective response rate, and the study was stopped in the population due to disease progression and increased side effects. In the phase III trial (NCT00861614), the 1-year survival rate for PCa patients treated with ipilimumab was 46.5%, compared to 40.8% in the placebo group, a difference that was not as significant as expected. These results suggest that immunotherapy is not yet mature in the treatment of PCa. These results highlight the need for careful patient selection for ICPI to identify subgroups of patients who may benefit from this treatment approach. Some studies have shown that the expression level of PD-L1 will also increase in CRPC treated with enzalutamide, suggesting that ADT may activate adaptive immunity (26). Another study has shown that ADT combined with abiraterone and prednisone does not increase the expression level of PD-L1 in PCa, and the response to ICPI is not ideal (27). Therefore, more studies are needed to improve the association between ADT and PD-1/PD-L1 expression levels. The results of higher PD-L1 expression in the “ICD-H- group C-low risk” group are consistent with our hypothesis that “ICD response alters the PCa immune microenvironment to attract more T cells”. In addition, we noted that although there was no difference in PD-1 expression between the ICD-H and ICD-L, the group C population had a significantly higher PD-1 expression, suggesting that group C may benefit from anti-PD-1 and anti-CTLA-4 therapies. This indicates that the PCa population needs to be further subdivided, which should not stop at ICD-H and ICD-L. ICD response will bring a high level of immune response and improve the prognosis of patients, which is also the logic of ICD-H as a high level of ICD response. Therefore, in the end, for the eight CRPC progression-related factors found by machine learning, in addition to being used to assist in judging the progression of the patient’s disease state, the most important thing is that these factors are closely related to the four risk genes, which indicates the accuracy of risk genes.

There are some limitations in our research. On the one hand, the prognosis models are acquired from TCGA, GEO, and MSKCC. To confirm the predictive significance of this prognostic signature, large-scale prospective clinical research is required. On the other hand, we revealed a four-risk-gene prognostic signature for PCa, but this is only a bioinformatics analysis, which lacks relevant experimental verification. Lastly, the level of ICD response in PCa still needs to be further verified by laboratory work, and the most direct evidence of ICD is the activation of DAMPs. Although we also found a potential association between CST2 and DAMPs, it is not the most direct evidence.

## 5 Conclusion

In summary, we successfully separated 901 samples into two subtypes (ICD-H and ICD-L) on the basis of the expression of the ICD-related genes and developed a prognostic RS involving four genes derived from the DEGs between the two subtypes. The RS can potentially be applied to determine prognosis, dendritic cell infiltration, expression levels of CRPC genes and ICD-related genes, and the function of immune-related pathways in prostate cancer. The ICD-H population may have a better response to ICPIs, and according to the RS calculation, this group has a lower risk. We further constructed a survival-predicting nomogram that combines this signature with other commonly used clinicopathological characteristics. These results can provide clinicians with a more quantitative approach to objectively predict survival rates for patients with prostate cancer.

## Data availability statement

The original contributions presented in the study are included in the article/**Supplementary Material**. Further inquiries can be directed to the corresponding authors.

## Author contributions

ZK and YW designed the study. ZK and J-BS wrote the manuscript. NX, D-NC, and Q-SZ collected relevant data and information. X-YH, FL, QH, and X-YX analyzed the data. All authors approved the submitted version. YW is the first correspondence author. NX is the co-correspondence author. All authors contributed to the article and approved the submitted version.
