# Supplementary material for: Subtype and prognostic analysis of immunogenic cell death-related gene signature in prostate cancer
Source: Front Oncol. 2023 Jun 6;13:1160972. doi: 10.3389/fonc.2023.1160972 (PMC10279955; doi:10.3389/fonc.2023.1160972)
Supplement: Supplementary file 1 [file DataSheet_1.doc]

**Some of the figures in this paper are derived from R language. The following is the related code and analysis process, and the input files used can be obtained from the link** *https://www.jianguoyun.com/p/DdbXIuAQpv21Cxif7vcEIAA***.**

**Figure 1 Related code is as follows:**

library(maftools)

setwd("")

geneRT=read.table("1.txt", header=T, sep="\t", check.names=F, row.names=1)

gene=row.names(geneRT)

pdf(file="Figure1A.pdf", width=8, height=7.5)

maf=read.maf(maf="2.maf")

oncoplot(maf=maf, genes=gene, fontSize=0.5, draw_titv=T)

dev.off()

library("RCircos")

setwd("")

cytoBandIdeogram=read.table("3.txt", header=T, sep="\t")

chr.exclude <- NULL

cyto.info <- cytoBandIdeogram

tracks.inside <- 5

tracks.outside <- 0

RCircos.Set.Core.Components(cyto.info, chr.exclude, tracks.inside, tracks.outside)

rcircos.params <- RCircos.Get.Plot.Parameters()

rcircos.params$text.size=0.8

rcircos.params$point.size=5

RCircos.Reset.Plot.Parameters(rcircos.params)

pdf(file="Figure1B.pdf", width=8, height=8)

RCircos.Set.Plot.Area()

RCircos.Chromosome.Ideogram.Plot()

RCircos.Scatter.Data=read.table("Rcircos.scatter.txt", header=T, sep="\t", check.names=F)

data.col <- 4

track.num <- 1

side <- "in"

RCircos.Scatter.Plot(RCircos.Scatter.Data, data.col, track.num, side, by.fold=0.1)

RCircos.Gene.Label.Data=read.table("Rcircos.geneLabel.txt", header=T, sep="\t", check.names=F)

name.col <- 4

side <- "in"

track.num <- 2

RCircos.Gene.Connector.Plot(RCircos.Gene.Label.Data, track.num, side)

track.num <- 3

RCircos.Gene.Name.Plot(RCircos.Gene.Label.Data, name.col, track.num, side)

dev.off()

inputFile="4.txt"

setwd("")

rt=read.table(inputFile, header=T, sep="\t", check.names=F, row.names=1)

GAIN=rowSums(rt> 0)

LOSS=rowSums(rt< 0)

GAIN=GAIN/ncol(rt)*100

LOSS=LOSS/ncol(rt)*100

data=cbind(GAIN, LOSS)

data=data[order(data[,"GAIN"],decreasing = T),]

data.max = apply(data, 1, max)

pdf(file="Figure1C.pdf", width=9, height=6)

cex=1.3

par(cex.lab=cex, cex.axis=cex, font.axis=2, las=1, xpd=T)

bar=barplot(data.max, col="grey80", border=NA,

xlab="", ylab="CNV.frequency(%)", space=1.5,

xaxt="n", ylim=c(0,1.2*max(data.max)))

points(bar,data[,"GAIN"], pch=20, col=2, cex=3)

points(bar,data[,"LOSS"], pch=20, col=3, cex=3)

legend("top", legend=c('GAIN','LOSS'), col=2:3, pch=20, bty="n", cex=2, ncol=2)

par(srt=45)

text(bar, par('usr')[3]-0.2, rownames(data), adj=1, cex=0.7)

dev.off()

library(limma)

library(reshape2)

library(ggpubr)

expFile="5.txt"

geneFile="1.txt"

setwd("")

rt=read.table(expFile, header=T, sep="\t", check.names=F)

rt=as.matrix(rt)

rownames(rt)=rt[,1]

exp=rt[,2:ncol(rt)]

dimnames=list(rownames(exp), colnames(exp))

data=matrix(as.numeric(as.matrix(exp)), nrow=nrow(exp), dimnames=dimnames)

data=avereps(data)

gene=read.table(geneFile, header=T, sep="\t", check.names=F)

sameGene=intersect(as.vector(gene[,1]), row.names(data))

data=data[sameGene,]

group=sapply(strsplit(colnames(data),"\\-"), "[", 4)

group=sapply(strsplit(group,""), "[", 1)

group=gsub("2", "1", group)

conNum=length(group[group==1])

treatNum=length(group[group==0])

sampleType=c(rep(1,conNum), rep(2,treatNum))

exp=log2(data+1)

exp=as.data.frame(t(exp))

exp=cbind(exp, Type=sampleType)

exp$Type=ifelse(exp$Type==1, "Normal", "Tumor")

sigGene=c()

for(i in colnames(exp)[1:(ncol(exp)-1)]){

if(sd(exp[,i])<0.001){next}

wilcoxTest=wilcox.test(exp[,i] ~ exp[,"Type"])

pvalue=wilcoxTest$p.value

if(wilcoxTest$p.value<0.05){

sigGene=c(sigGene, i)

}

}

sigGene=c(sigGene, "Type")

exp=exp[,sigGene]

data=melt(exp, id.vars=c("Type"))

colnames(data)=c("Type", "Gene", "Expression")

p=ggboxplot(data, x="Gene", y="Expression", color = "Type",

ylab="Gene expression",

xlab="",

legend.title="Type",

palette = c("blue", "red"),

width=1)

p=p+rotate_x_text(60)

p1=p+stat_compare_means(aes(group=Type),

method="wilcox.test",

symnum.args=list(cutpoints = c(0, 0.001, 0.01, 0.05, 1), symbols = c("***", "**", "*", " ")),

label = "p.signif")

pdf(file="Figure1D.pdf", width=9, height=6)

print(p1)

dev.off()

**Figure 2 Related code is as follows:**

library(ConsensusClusterPlus)

expFile="6.txt"

workDir=""

setwd(workDir)

data=read.table(expFile, header=T, sep="\t", check.names=F, row.names=1)

data=as.matrix(data)

maxK=9

results=ConsensusClusterPlus(data,

maxK=maxK,

reps=50,

pItem=0.8,

pFeature=1,

title=workDir,

clusterAlg="pam",

distance="euclidean",

seed=899776,

plot="png")

clusterNum=2

cluster=results[[clusterNum]][["consensusClass"]]

cluster=as.data.frame(cluster)

colnames(cluster)=c("ICDcluster")

letter=c("A","B","C","D","E","F","G")

uniqClu=levels(factor(cluster$ICDcluster))

cluster$ICDcluster=letter[match(cluster$ICDcluster, uniqClu)]

clusterOut=rbind(ID=colnames(cluster), cluster)

library(survival)

library(survminer)

clusterFile="7.txt"

cliFile="8.txt"

cluster=read.table(clusterFile, header=T, sep="\t", check.names=F, row.names=1)

rownames(cluster)=gsub("(.*?)\\_(.*?)", "\\2", rownames(cluster))

cli=read.table(cliFile, header=T, sep="\t", check.names=F, row.names=1)

colnames(cli)=c("futime", "fustat")

cli$futime=cli$futime/365

sameSample=intersect(row.names(cluster), row.names(cli))

rt=cbind(cli[sameSample,,drop=F], cluster[sameSample,,drop=F])

length=length(levels(factor(rt$ICDcluster)))

diff=survdiff(Surv(futime, fustat) ~ ICDcluster, data = rt)

pValue=1-pchisq(diff$chisq, df=length-1)

if(pValue<0.001){

pValue="p<0.001"

}else{

pValue=paste0("p=",sprintf("%.03f",pValue))

}

fit <- survfit(Surv(futime, fustat) ~ ICDcluster, data = rt)

#print(surv_median(fit))

bioCol=c("#0066FF","#FF9900","#FF0000","#6E568C","#7CC767","#223D6C","#D20A13","#FFD121","#088247","#11AA4D")

bioCol=bioCol[1:length]

surPlot=ggsurvplot(fit,

data=rt,

conf.int=F,

pval=pValue,

pval.size=6,

legend.title="ICDcluster",

legend.labs=levels(factor(rt[,"ICDcluster"])),

legend = c(0.8, 0.8),

font.legend=10,

xlab="Time(years)",

break.time.by = 1,

palette = bioCol,

surv.median.line = "hv",

risk.table=T,

cumevents=F,

risk.table.height=.25)

pdf(file="Figure2B.pdf",onefile = FALSE,width=7,height=5.5)

print(surPlot)

dev.off()

library(reshape2)

library(ggpubr)

library(limma)

library(GSEABase)

library(GSVA)

expFile="9.txt"

clusterFile="7.txt"

gmtFile="10.gmt"

rt=read.table(expFile, header=T, sep="\t", check.names=F)

rt=as.matrix(rt)

rownames(rt)=rt[,1]

exp=rt[,2:ncol(rt)]

dimnames=list(rownames(exp),colnames(exp))

data=matrix(as.numeric(as.matrix(exp)),nrow=nrow(exp),dimnames=dimnames)

data=avereps(data)

geneSets=getGmt(gmtFile, geneIdType=SymbolIdentifier())

ssgseaScore=gsva(data, geneSets, method='ssgsea', kcdf='Gaussian', abs.ranking=TRUE)

normalize=function(x){

return((x-min(x))/(max(x)-min(x)))}

ssgseaScore=normalize(ssgseaScore)

ssgseaOut=rbind(id=colnames(ssgseaScore), ssgseaScore)

write.table(ssgseaOut,file="ssGSEA.result.txt",sep="\t",quote=F,col.names=F)

cluster=read.table(clusterFile, header=T, sep="\t", check.names=F, row.names=1)

ssgseaScore=t(ssgseaScore)

sameSample=intersect(row.names(ssgseaScore), row.names(cluster))

ssgseaScore=ssgseaScore[sameSample,,drop=F]

cluster=cluster[sameSample,,drop=F]

scoreCluster=cbind(ssgseaScore, cluster)

data=melt(scoreCluster, id.vars=c("ICDcluster"))

colnames(data)=c("ICDcluster", "Immune", "Fraction")

bioCol=c("#0066FF","#FF9900","#FF0000","#6E568C","#7CC767","#223D6C","#D20A13","#FFD121","#088247","#11AA4D")

bioCol=bioCol[1:length(levels(factor(data[,"ICDcluster"])))]

p=ggboxplot(data, x="Immune", y="Fraction", color="ICDcluster",

ylab="Immune infiltration",

xlab="",

legend.title="ICDcluster",

palette=bioCol)

p=p+rotate_x_text(50)

pdf(file="Figure2C.pdf", width=8, height=6.5)

p+stat_compare_means(aes(group=ICDcluster),symnum.args=list(cutpoints = c(0, 0.001, 0.01, 0.05, 1), symbols = c("***", "**", "*", "")),label = "p.signif")

dev.off()

library(pheatmap)

expFile="4.txt"

clusterFile="7.txt"

cliFile="11.txt"

exp=read.table(expFile, header=T, sep="\t", check.names=F, row.names=1)

exp=t(exp)

cluster=read.table(clusterFile, header=T, sep="\t", check.names=F, row.names=1)

sameSample=intersect(row.names(exp), row.names(cluster))

exp=exp[sameSample, , drop=F]

cluster=cluster[sameSample, , drop=F]

expCluster=cbind(exp, cluster)

Project=gsub("(.*?)\\_.*", "\\1", rownames(expCluster))

rownames(expCluster)=gsub("(.*?)\\_(.*?)", "\\2", rownames(expCluster))

expCluster=cbind(expCluster, Project)

cli=read.table(cliFile, header=T, sep="\t", check.names=F, row.names=1)

cli[,"Age"]=ifelse(cli[,"Age"]=="unknow", "unknow", ifelse(cli[,"Age"]>65,">65","<=65"))

sameSample=intersect(row.names(expCluster), row.names(cli))

expCluster=expCluster[sameSample,,drop=F]

cli=cli[sameSample,,drop=F]

data=cbind(expCluster, cli)

data=data[order(data$ICDcluster),]

Type=data[,((ncol(exp)+1):ncol(data))]

data=t(data[,1:ncol(exp)])

bioCol=c("#0066FF","#FF9900","#FF0000","#6E568C","#7CC767","#223D6C","#D20A13","#FFD121","#088247","#11AA4D")

ann_colors=list()

ICDCluCol=bioCol[1:length(levels(factor(Type$ICDcluster)))]

names(ICDCluCol)=levels(factor(Type$ICDcluster))

ann_colors[["ICDcluster"]]=ICDCluCol

pdf("Figure2D.pdf", width=7.5, height=5)

pheatmap(data,

annotation=Type,

annotation_colors = ann_colors,

color = colorRampPalette(c(rep("blue",5), "white", rep("red",5)))(100),

cluster_cols =F,

cluster_rows =F,

scale="row",

show_colnames=F,

fontsize=6,

fontsize_row=6,

fontsize_col=6)

dev.off()

library(limma)

library(GSEABase)

library(GSVA)

library(pheatmap)

expFile="10.txt"

clusterFile="7.txt"

gmtFile="c2.cp.kegg.v7.4.symbols.gmt"

setwd("")

rt=read.table(expFile, header=T, sep="\t", check.names=F)

rt=as.matrix(rt)

rownames(rt)=rt[,1]

exp=rt[,2:ncol(rt)]

dimnames=list(rownames(exp), colnames(exp))

data=matrix(as.numeric(as.matrix(exp)), nrow=nrow(exp), dimnames=dimnames)

data=avereps(data)

geneSets=getGmt(gmtFile, geneIdType=SymbolIdentifier())

gsvaResult=gsva(data,

geneSets,

min.sz=10,

max.sz=500,

verbose=TRUE,

parallel.sz=1)

gsvaOut=rbind(id=colnames(gsvaResult), gsvaResult)

write.table(gsvaOut, file="gsvaOut.txt", sep="\t", quote=F, col.names=F)

cluster=read.table(clusterFile, header=T, sep="\t", check.names=F, row.names=1)

gsvaResult=t(gsvaResult)

sameSample=intersect(row.names(gsvaResult), row.names(cluster))

gsvaResult=gsvaResult[sameSample,,drop=F]

cluster=cluster[sameSample,,drop=F]

gsvaCluster=cbind(gsvaResult, cluster)

Project=gsub("(.*?)\\_.*", "\\1", rownames(gsvaCluster))

gsvaCluster=cbind(gsvaCluster, Project)

adj.P.Val.Filter=0.05

allType=as.vector(gsvaCluster$ICDcluster)

comp=combn(levels(factor(allType)), 2)

for(i in 1:ncol(comp)){

treat=gsvaCluster[gsvaCluster$ICDcluster==comp[2,i],]

con=gsvaCluster[gsvaCluster$ICDcluster==comp[1,i],]

data=rbind(con, treat)

Type=as.vector(data$ICDcluster)

ann=data[,c(ncol(data), (ncol(data)-1))]

data=t(data[,-c((ncol(data)-1), ncol(data))])

design=model.matrix(~0+factor(Type))

colnames(design)=levels(factor(Type))

fit=lmFit(data, design)

contrast=paste0(comp[2,i], "-", comp[1,i])

cont.matrix=makeContrasts(contrast, levels=design)

fit2=contrasts.fit(fit, cont.matrix)

fit2=eBayes(fit2)

allDiff=topTable(fit2,adjust='fdr',number=200000)

allDiffOut=rbind(id=colnames(allDiff),allDiff)

write.table(allDiffOut, file=paste0(contrast, ".all.txt"), sep="\t", quote=F, col.names=F)

diffSig=allDiff[with(allDiff, (abs(logFC)>0.1 & adj.P.Val < adj.P.Val.Filter )), ]

diffSigOut=rbind(id=colnames(diffSig),diffSig)

write.table(diffSigOut, file=paste0(contrast, ".diff.txt"), sep="\t", quote=F, col.names=F)

bioCol=c("#0066FF","#FF9900","#FF0000","#6E568C","#7CC767","#223D6C","#D20A13","#FFD121","#088247","#11AA4D")

ann_colors=list()

m6aCluCol=bioCol[1:length(levels(factor(allType)))]

names(m6aCluCol)=levels(factor(allType))

ann_colors[["ICDcluster"]]=m6aCluCol[c(comp[1,i], comp[2,i])]

termNum=20

diffTermName=as.vector(rownames(diffSig))

diffLength=length(diffTermName)

if(diffLength<termNum){termNum=diffLength}

hmGene=diffTermName[1:termNum]

hmExp=data[hmGene,]

pdf(file=paste0(contrast,".Figure2E.pdf"), width=10, height=6)

pheatmap(hmExp,

annotation=ann,

annotation_colors = ann_colors,

color = colorRampPalette(c(rep("blue",2), "white", rep("red",2)))(50),

cluster_cols =F,

show_colnames = F,

gaps_col=as.vector(cumsum(table(Type))),

scale="row",

fontsize = 8,

fontsize_row=6,

fontsize_col=8)

dev.off()

}

library(limma)

library(VennDiagram)

expFile="10.txt"

cluFile="7.txt"

logFCfilter=0.585

adj.P.Val.Filter=0.05

rt=read.table(expFile, header=T, sep="\t", check.names=F)

rt=as.matrix(rt)

rownames(rt)=rt[,1]

exp=rt[,2:ncol(rt)]

dimnames=list(rownames(exp),colnames(exp))

data=matrix(as.numeric(as.matrix(exp)),nrow=nrow(exp),dimnames=dimnames)

data=avereps(data)

data=data[rowMeans(data)>0,]

cluster=read.table(cluFile, header=T, sep="\t", check.names=F, row.names=1)

sameSample=intersect(colnames(data), row.names(cluster))

data=data[,sameSample]

cluster=cluster[sameSample,]

geneList=list()

Type=as.vector(cluster)

design=model.matrix(~0+factor(Type))

colnames(design)=levels(factor(Type))

comp=combn(levels(factor(Type)), 2)

allDiffGenes=c()

for(i in 1:ncol(comp)){

fit=lmFit(data, design)

contrast=paste0(comp[2,i], "-", comp[1,i])

#print(contrast)

cont.matrix=makeContrasts(contrast, levels=design)

fit2=contrasts.fit(fit, cont.matrix)

fit2=eBayes(fit2)

allDiff=topTable(fit2,adjust='fdr',number=200000)

allDiffOut=rbind(id=colnames(allDiff),allDiff)

write.table(allDiffOut, file=paste0(contrast, ".all.txt"), sep="\t", quote=F, col.names=F)

diffSig=allDiff[with(allDiff, (abs(logFC)>logFCfilter & adj.P.Val < adj.P.Val.Filter )), ]

diffSigOut=rbind(id=colnames(diffSig),diffSig)

write.table(diffSigOut, file=paste0(contrast, ".diff.txt"), sep="\t", quote=F, col.names=F)

geneList[[contrast]]=row.names(diffSig)

}

venn.plot=venn.diagram(geneList,filename=NULL,fill=rainbow(length(geneList)) )

pdf(file="venn.pdf", width=5, height=5)

grid.draw(Figure2F.plot)

dev.off()

**Figure 3 Related code is as follows:**

library(limma)

library(survival)

expFile="12.txt"

cliFile="8.txt"

rt=read.table(expFile, header=T, sep="\t", check.names=F)

rt=as.matrix(rt)

rownames(rt)=rt[,1]

exp=rt[,2:ncol(rt)]

dimnames=list(rownames(exp), colnames(exp))

data=matrix(as.numeric(as.matrix(exp)), nrow=nrow(exp), dimnames=dimnames)

data=avereps(data)

data=data[rowMeans(data)>0,]

data1=t(data)

rownames(data1)=gsub("(.*?)\\_(.*?)", "\\2", rownames(data1))

cli=read.table(cliFile, header=T, sep="\t", check.names=F, row.names=1) #??ȡ?ٴ??ļ?

cli$futime=cli$futime/365

sameSample=intersect(row.names(data1), row.names(cli))

data1=data1[sameSample,,drop=F]

cli=cli[sameSample,,drop=F]

rt=cbind(cli, data1)

outTab=data.frame()

sigGenes=c()

for(i in colnames(rt[,3:ncol(rt)])){

#cox????

cox <- coxph(Surv(futime, fustat) ~ rt[,i], data = rt)

coxSummary = summary(cox)

coxP=coxSummary$coefficients[,"Pr(>|z|)"]

if(coxP<0.05){

sigGenes=c(sigGenes,i)

outTab=rbind(outTab,

cbind(id=i,

HR=coxSummary$conf.int[,"exp(coef)"],

HR.95L=coxSummary$conf.int[,"lower .95"],

HR.95H=coxSummary$conf.int[,"upper .95"],

pvalue=coxSummary$coefficients[,"Pr(>|z|)"])

)

}

}

write.table(outTab,file="Figure3A.txt",sep="\t",row.names=F,quote=F)

library(limma)

library(ConsensusClusterPlus)

expFile="13.txt"

rt=read.table(expFile, header=T, sep="\t", check.names=F)

rt=as.matrix(rt)

rownames(rt)=rt[,1]

exp=rt[,2:ncol(rt)]

dimnames=list(rownames(exp), colnames(exp))

data=matrix(as.numeric(as.matrix(exp)), nrow=nrow(exp), dimnames=dimnames)

data=avereps(data)

data=data[rowMeans(data)>0,]

maxK=9

results=ConsensusClusterPlus(data,

maxK=maxK,

reps=50,

pItem=0.8,

pFeature=1,

title=workDir,

clusterAlg="km",

distance="euclidean",

seed=123456,

plot="png")

clusterNum=3

cluster=results[[clusterNum]][["consensusClass"]]

cluster=as.data.frame(cluster)

colnames(cluster)=c("geneCluster")

letter=c("A","B","C","D","E","F","G")

uniqClu=levels(factor(cluster$geneCluster))

cluster$geneCluster=letter[match(cluster$geneCluster, uniqClu)]

clusterOut=rbind(ID=colnames(cluster), cluster)

library(survival)

library(survminer)

clusterFile="14.txt"

cliFile="8.txt"

cluster=read.table(clusterFile, header=T, sep="\t", check.names=F, row.names=1)

rownames(cluster)=gsub("(.*?)\\_(.*?)", "\\2", rownames(cluster))

cli=read.table(cliFile, header=T, sep="\t", check.names=F, row.names=1)

colnames(cli)=c("futime", "fustat")

cli$futime=cli$futime/365

sameSample=intersect(row.names(cluster), row.names(cli))

rt=cbind(cli[sameSample,,drop=F], cluster[sameSample,,drop=F])

length=length(levels(factor(rt$geneCluster)))

diff=survdiff(Surv(futime, fustat) ~ geneCluster, data = rt)

pValue=1-pchisq(diff$chisq, df=length-1)

if(pValue<0.001){

pValue="p<0.001"

}else{

pValue=paste0("p=",sprintf("%.03f",pValue))

}

fit <- survfit(Surv(futime, fustat) ~ geneCluster, data = rt)

bioCol=c("#0066FF","#FF9900","#FF0000","#6E568C","#7CC767","#223D6C","#D20A13","#FFD121","#088247","#11AA4D")

bioCol=bioCol[1:length(levels(factor(rt[,"geneCluster"])))]

surPlot=ggsurvplot(fit,

data=rt,

conf.int=F,

pval=pValue,

pval.size=6,

legend.title="geneCluster",

legend.labs=levels(factor(rt[,"geneCluster"])),

legend = c(0.8, 0.8),

font.legend=10,

xlab="Time(years)",

break.time.by = 1,

palette = bioCol,

surv.median.line = "hv",

risk.table=T,

cumevents=F,

risk.table.height=.25)

pdf(file="Figure3C.pdf", onefile = FALSE, width=7, height=5.5)

print(surPlot)

dev.off()

expFile="13.txt"

geneCluFile="14.txt"

ICDCluFile="7.txt"

cliFile="11.txt"

exp=read.table(expFile, header=T, sep="\t", check.names=F, row.names=1)

ICDClu=read.table(ICDCluFile, header=T, sep="\t", check.names=F, row.names=1)

geneClu=read.table(geneCluFile, header=T, sep="\t", check.names=F, row.names=1)

exp=as.data.frame(t(exp))

sameSample=intersect(row.names(exp), row.names(ICDClu))

exp=exp[sameSample,,drop=F]

expData=cbind(exp, geneCluster=geneClu[sameSample,], ICDcluster=ICDClu[sameSample,])

Project=gsub("(.*?)\\_.*", "\\1", rownames(expData))

rownames(expData)=gsub("(.*?)\\_(.*?)", "\\2", rownames(expData))

expData=cbind(expData, Project)

cli=read.table(cliFile, header=T, sep="\t", check.names=F, row.names=1)

cli[,"Age"]=ifelse(cli[,"Age"]=="unknow", "unknow", ifelse(cli[,"Age"]>65,">65","<=65"))

sameSample=intersect(row.names(expData), row.names(cli))

expData=expData[sameSample,,drop=F]

cli=cli[sameSample,,drop=F]

data=cbind(expData, cli)

data=data[order(data$geneCluster),]

Type=data[,((ncol(data)-2-ncol(cli)):ncol(data))]

data=t(data[,1:(ncol(expData)-3)])

bioCol=c("#0066FF","#FF9900","#FF0000","#6E568C","#7CC767","#223D6C","#D20A13","#FFD121","#088247","#11AA4D")

ann_colors=list()

ICDcol=bioCol[1:length(levels(factor(Type$ICDcluster)))]

names(ICDcol)=levels(factor(Type$ICDcluster))

ann_colors[["ICDcluster"]]=ICDcol

GENEcol=bioCol[1:length(levels(factor(Type$geneCluster)))]

names(GENEcol)=levels(factor(Type$geneCluster))

ann_colors[["geneCluster"]]=GENEcol

pdf("Figure3D.pdf", height=6, width=8)

pheatmap(data,

annotation=Type,

annotation_colors = ann_colors,

color = colorRampPalette(c(rep("blue",5), "white", rep("red",5)))(50),

cluster_cols =F,

cluster_rows =F,

scale="row",

show_colnames=F,

show_rownames=F,

fontsize=6,

fontsize_row=2,

fontsize_col=6)

dev.off()

library(limma)

library(reshape2)

library(ggpubr)

expFile="7.txt"

geneCluFile="14.txt"

rt=read.table(expFile, header=T, sep="\t", check.names=F)

rt=as.matrix(rt)

rownames(rt)=rt[,1]

exp=rt[,2:ncol(rt)]

dimnames=list(rownames(exp),colnames(exp))

data=matrix(as.numeric(as.matrix(exp)),nrow=nrow(exp),dimnames=dimnames)

data=avereps(data)

data=t(data)

geneClu=read.table(geneCluFile, header=T, sep="\t", check.names=F, row.names=1)

sameSample=intersect(row.names(data), row.names(geneClu))

expClu=cbind(data[sameSample,,drop=F], geneClu[sameSample,,drop=F])

sigGene=c()

for(i in colnames(expClu)[1:(ncol(expClu)-1)]){

if(sd(expClu[,i])<0.001){next}

if(length(levels(factor(expClu[,"geneCluster"])))>2){

test=kruskal.test(expClu[,i] ~ expClu[,"geneCluster"])

}else{

test=wilcox.test(expClu[,i] ~ expClu[,"geneCluster"])

}

pvalue=test$p.value

if(pvalue<0.05){

sigGene=c(sigGene, i)

}

}

sigGene=c(sigGene, "geneCluster")

expClu=expClu[,sigGene]

data=melt(expClu, id.vars=c("geneCluster"))

colnames(data)=c("geneCluster", "Gene", "Expression")

bioCol=c("#0066FF","#FF9900","#FF0000","#6E568C","#7CC767","#223D6C","#D20A13","#FFD121","#088247","#11AA4D")

bioCol=bioCol[1:length(levels(factor(data[,"geneCluster"])))]

p=ggboxplot(data, x="Gene", y="Expression", color = "geneCluster",

xlab="",

ylab="Gene expression",

legend.title="geneCluster",

palette = bioCol,

width=1)

p=p+rotate_x_text(60)

p1=p+stat_compare_means(aes(group=geneCluster),

symnum.args=list(cutpoints = c(0, 0.001, 0.01, 0.05, 1), symbols = c("***", "**", "*", " ")),

label = "p.signif")

pdf(file="Figure3E.pdf", width=9, height=6)

print(p1)

dev.off()

#???ð?

library(survival)

library(caret)

library(glmnet)

library(survminer)

library(timeROC)

rt=read.table("15.txt", header=T, sep="\t", check.names=F, row.names=1) rt$futime[rt$futime<=0]=0.003

n=100

for(i in 1:n){

inTrain<-createDataPartition(y=rt[,2], p=0.5, list=F)

train<-rt[inTrain,]

test<-rt[-inTrain,]

trainOut=cbind(id=row.names(train),train)

testOut=cbind(id=row.names(test),test)

x=as.matrix(train[,c(3:ncol(train))])

y=data.matrix(Surv(train$futime,train$fustat))

fit <- glmnet(x, y, family = "cox", maxit = 1000)

cvfit <- cv.glmnet(x, y, family="cox", maxit = 1000)

coef <- coef(fit, s = cvfit$lambda.min)

index <- which(coef != 0)

actCoef <- coef[index]

lassoGene=row.names(coef)[index]

lassoSigExp=train[,c("futime", "fustat", lassoGene)]

lassoSigExpOut=cbind(id=row.names(lassoSigExp), lassoSigExp)

geneCoef=cbind(Gene=lassoGene, Coef=actCoef)

if(nrow(geneCoef)<2){next}

multiCox <- coxph(Surv(futime, fustat) ~ ., data = lassoSigExp)

multiCox=step(multiCox,direction = "both")

multiCoxSum=summary(multiCox)

outMultiTab=data.frame()

outMultiTab=cbind(

coef=multiCoxSum$coefficients[,"coef"],

HR=multiCoxSum$conf.int[,"exp(coef)"],

HR.95L=multiCoxSum$conf.int[,"lower .95"],

HR.95H=multiCoxSum$conf.int[,"upper .95"],

pvalue=multiCoxSum$coefficients[,"Pr(>|z|)"])

outMultiTab=cbind(id=row.names(outMultiTab),outMultiTab)

outMultiTab=outMultiTab[,1:2]

riskScore=predict(multiCox,type="risk",newdata=train)

coxGene=rownames(multiCoxSum$coefficients)

coxGene=gsub("`","",coxGene)

outCol=c("futime","fustat",coxGene)

medianTrainRisk=median(riskScore)

risk=as.vector(ifelse(riskScore>medianTrainRisk,"high","low"))

trainRiskOut=cbind(id=rownames(cbind(train[,outCol],riskScore,risk)),cbind(train[,outCol],riskScore,risk))

riskScoreTest=predict(multiCox,type="risk",newdata=test) #????train?õ?ģ??Ԥ??test??Ʒ????

riskTest=as.vector(ifelse(riskScoreTest>medianTrainRisk,"high","low"))

testRiskOut=cbind(id=rownames(cbind(test[,outCol],riskScoreTest,riskTest)),cbind(test[,outCol],riskScore=riskScoreTest,risk=riskTest))

diff=survdiff(Surv(futime, fustat) ~risk,data = train)

pValue=1-pchisq(diff$chisq, df=1)

diffTest=survdiff(Surv(futime, fustat) ~riskTest,data = test)

pValueTest=1-pchisq(diffTest$chisq, df=1)

predictTime=3

roc=timeROC(T=train$futime, delta=train$fustat,

marker=riskScore, cause=1,

times=c(predictTime), ROC=TRUE)

rocTest=timeROC(T=test$futime, delta=test$fustat,

marker=riskScoreTest, cause=1,

times=c(predictTime), ROC=TRUE)

if((pValue<0.01) & (roc$AUC[2]>0.65) & (pValueTest<0.05) & (rocTest$AUC[2]>0.63)){

write.table(trainOut,file="data.train.txt",sep="\t",quote=F,row.names=F)

write.table(testOut,file="data.test.txt",sep="\t",quote=F,row.names=F)

#lasso????

write.table(lassoSigExpOut,file="lasso.SigExp.txt",sep="\t",row.names=F,quote=F)

pdf("Figure3G.pdf")

plot(fit, xvar = "lambda", label = TRUE)

dev.off()

pdf("Figure3F.pdf")

plot(cvfit)

abline(v=log(c(cvfit$lambda.min,cvfit$lambda.1se)), lty="dashed")

dev.off()

write.table(outMultiTab,file="multiCox.txt",sep="\t",row.names=F,quote=F)

write.table(trainRiskOut,file="risk.train.txt",sep="\t",quote=F,row.names=F)

write.table(testRiskOut,file="risk.test.txt",sep="\t",quote=F,row.names=F)

allRiskOut=rbind(trainRiskOut, testRiskOut)

write.table(allRiskOut,file="risk.all.txt",sep="\t",quote=F,row.names=F)

break

}

}

**Figure 4 Related code is as follows:**

library(survival)

library(survminer)

bioSurvival=function(inputFile=null, outFile=null){

rt=read.table(inputFile, header=T, sep="\t", check.names=F)

diff=survdiff(Surv(futime, fustat) ~risk,data = rt)

pValue=1-pchisq(diff$chisq,df=1)

if(pValue<0.001){

pValue="p<0.001"

}else{

pValue=paste0("p=",sprintf("%.03f",pValue))

}

fit <- survfit(Surv(futime, fustat) ~ risk, data = rt)

surPlot=ggsurvplot(fit,

data=rt,

conf.int=T,

pval=pValue,

pval.size=6,

legend.title="risk",

legend.labs=c("High risk", "Low risk"),

xlab="Time(years)",

break.time.by = 1,

palette=c("red", "blue"),

risk.table=TRUE,

risk.table.title="",

risk.table.col = "strata",

risk.table.height=.25)

pdf(file=outFile,onefile = FALSE,width = 6.5,height =5.5)

print(surPlot)

dev.off()

}

bioSurvival(inputFile="16.txt", outFile="Figure4A.pdf")

bioSurvival(inputFile="17.txt", outFile="Figure4B.pdf")

bioROC=function(inputFile=null, rocFile=null){

rt=read.table(inputFile, header=T, sep="\t", check.names=F)

ROC_rt=timeROC(T=rt$futime,delta=rt$fustat,

marker=rt$riskScore,cause=1,

weighting='aalen',

times=c(1,3,5),ROC=TRUE)

pdf(file=rocFile, width=5, height=5)

plot(ROC_rt,time=1,col='green',title=FALSE,lwd=2)

plot(ROC_rt,time=3,col='blue',add=TRUE,title=FALSE,lwd=2)

plot(ROC_rt,time=5,col='red',add=TRUE,title=FALSE,lwd=2)

legend('bottomright',

c(paste0('AUC at 1 years: ',sprintf("%.03f",ROC_rt$AUC[1])),

paste0('AUC at 3 years: ',sprintf("%.03f",ROC_rt$AUC[2])),

paste0('AUC at 5 years: ',sprintf("%.03f",ROC_rt$AUC[3]))),

col=c("green",'blue','red'),lwd=2,bty = 'n')

dev.off()

}

bioROC(inputFile="16.txt", rocFile="Figure4C.pdf")

bioROC(inputFile="17.txt", rocFile="Figure4D.pdf")

library(survival)

library(survminer)

library(timeROC)

library(rms)

library(regplot)

riskFile="16.txt"

cliFile="11.txt"

risk=read.table(riskFile, header=T, sep="\t", check.names=F, row.names=1)

risk=risk[,c("futime", "fustat", "risk")]

cli=read.table(cliFile, header=T, sep="\t", check.names=F, row.names=1)

cli=na.omit(cli)

cli=cli[apply(cli,1,function(x)any(is.na(match('unknow',x)))),,drop=F]

samSample=intersect(row.names(risk), row.names(cli))

risk1=risk[samSample,,drop=F]

cli=cli[samSample,,drop=F]

rt=cbind(risk1, cli)

res.cox=coxph(Surv(futime, fustat) ~ . , data = rt)

nom1=regplot(res.cox,

plots = c("density", "boxes"),

clickable=F,

title="",

points=TRUE,

droplines=TRUE,

observation=rt[1,],

rank="sd",

failtime = c(1,3,5),

prfail = T)

nomoRisk=predict(res.cox, data=rt, type="risk")

rt$nomoRisk=nomoRisk

ROC_rt=timeROC(T=rt$futime, delta=rt$fustat,

marker=rt$nomoRisk, cause=1,

weighting='aalen',

times=c(1,3,5), ROC=TRUE)

pdf(file="Figure4G.pdf", width=5, height=5)

plot(ROC_rt,time=1,col='green',title=FALSE,lwd=2)

plot(ROC_rt,time=3,col='blue',add=TRUE,title=FALSE,lwd=2)

plot(ROC_rt,time=5,col='red',add=TRUE,title=FALSE,lwd=2)

legend('bottomright',

c(paste0('AUC at 1 years: ',sprintf("%.03f",ROC_rt$AUC[1])),

paste0('AUC at 3 years: ',sprintf("%.03f",ROC_rt$AUC[2])),

paste0('AUC at 5 years: ',sprintf("%.03f",ROC_rt$AUC[3]))),

col=c("green","blue","red"),lwd=2,bty = 'n')

dev.off()

pdf(file="Figure4H.pdf", width=5, height=5)

f <- cph(Surv(futime, fustat) ~ nomoRisk, x=T, y=T, surv=T, data=rt, time.inc=1)

cal <- calibrate(f, cmethod="KM", method="boot", u=1, m=(nrow(rt)/3), B=1000)

plot(cal, xlim=c(0,1), ylim=c(0,1),

xlab="Nomogram-predicted OS (%)", ylab="Observed OS (%)", lwd=1.5, col="green", sub=F)

f <- cph(Surv(futime, fustat) ~ nomoRisk, x=T, y=T, surv=T, data=rt, time.inc=3)

cal <- calibrate(f, cmethod="KM", method="boot", u=3, m=(nrow(rt)/3), B=1000)

plot(cal, xlim=c(0,1), ylim=c(0,1), xlab="", ylab="", lwd=1.5, col="blue", sub=F, add=T)

f <- cph(Surv(futime, fustat) ~ nomoRisk, x=T, y=T, surv=T, data=rt, time.inc=5)

cal <- calibrate(f, cmethod="KM", method="boot", u=5, m=(nrow(rt)/3), B=1000)

plot(Figure4F, xlim=c(0,1), ylim=c(0,1), xlab="", ylab="", lwd=1.5, col="red", sub=F, add=T)

legend('bottomright', c('1-year', '3-year', '5-year'),

col=c("green","blue","red"), lwd=1.5, bty = 'n')

dev.off()

library(limma)

library(reshape2)

library(tidyverse)

library(ggplot2)

library(ggpubr)

library(ggExtra)

immFile="18.txt"

riskFile="19l.txt"

immune=read.table(immFile, header=T, sep="\t", check.names=F, row.names=1)

immune=immune[immune[,"P-value"]<0.05,]

data=as.matrix(immune[,1:(ncol(immune)-3)])

rownames(data)=gsub("(.*?)\\_(.*?)", "\\2", rownames(data))

risk=read.table(riskFile, header=T, sep="\t", check.names=F, row.names=1)

sameSample=intersect(row.names(data), row.names(risk))

data=data[sameSample,,drop=F]

risk=risk[sameSample,,drop=F]

for(i in colnames(data)[1:ncol(data)]){

x=as.numeric(risk[,"riskScore"])

x[x>quantile(x,0.99)]=quantile(x,0.99)

y=as.numeric(data[,i])

if(sd(y)<0.01){next}

cor=cor.test(x, y, method="spearma")

if(cor$p.value<0.05){

outFile=paste0("cor.", i, ".pdf")

df1=as.data.frame(cbind(x,y))

p1=ggplot(df1, aes(x, y)) +

xlab("Risk score") + ylab(i)+

geom_point() + geom_smooth(method="lm",formula = y ~ x) + theme_bw()+

stat_cor(method = 'spearman', aes(x =x, y =y))

p2=ggMarginal(p1, type="density", xparams=list(fill = "orange"), yparams=list(fill = "blue"))

#??????ͼ??

pdf(file=outFile, width=5.2, height=5)

print(p2)

dev.off()

}

}

outTab=data.frame()

risk=risk[,3:(ncol(risk)-2),drop=F]

for(immune in colnames(data)){

for(gene in colnames(risk)){

x=as.numeric(data[,immune])

y=as.numeric(risk[,gene])

corT=cor.test(x,y,method="spearman")

cor=corT$estimate

pvalue=corT$p.value

text=ifelse(pvalue<0.001,"***",ifelse(pvalue<0.01,"**",ifelse(pvalue<0.05,"*","")))

outTab=rbind(outTab,cbind(Gene=gene, Immune=immune, cor, text, pvalue))

}

}

outTab$cor=as.numeric(outTab$cor)

pdf(file="Figure4L.pdf", width=7, height=6)

ggplot(outTab, aes(Gene, Immune)) +

geom_tile(aes(fill = cor), colour = "grey", size = 1)+

scale_fill_gradient2(low = "#5C5DAF", mid = "white", high = "#EA2E2D") +

geom_text(aes(label=text),col ="black",size = 3) +

theme_minimal() +

theme(axis.title.x=element_blank(), axis.ticks.x=element_blank(), axis.title.y=element_blank(),

axis.text.x = element_text(angle = 45, hjust = 1, size = 10, face = "bold"), #x??????

axis.text.y = element_text(size = 10, face = "bold")) + #y??????

labs(fill =paste0("*** p<0.001","\n", "** p<0.01","\n", " * p<0.05","\n", "\n","Correlation")) + #????ͼ??

scale_x_discrete(position = "bottom")

dev.off()

library(igraph)

library(psych)

library(reshape2)

library(RColorBrewer)

GeneExpfile <- "6.txt"

Genefile <- "1.txt"

Coxfile <- "20.txt"

gene.group <- read.table(Genefile,header=T,sep="\t")

gene.exp <- read.table(GeneExpfile,header=T,sep="\t",row.names=1)

gene.cox <- read.table(Coxfile,header=T,sep="\t")

colnames(gene.group) <- c('id','group')

genelist <- intersect(gene.group$id, gene.cox$id)

genelist <- intersect(genelist, rownames(gene.exp))

gene.group <- gene.group[match(genelist,gene.group$id),]

gene.group <- gene.group[order(gene.group$group),]

gene.exp <- gene.exp[match(gene.group$id,rownames(gene.exp)),]

gene.cox <- gene.cox[match(gene.group$id,gene.cox$id),]

gene.cor <- corr.test(t(gene.exp))

gene.cor.cor <- gene.cor$r

gene.cor.pvalue <- gene.cor$p

gene.cor.cor[upper.tri(gene.cor.cor)] = NA

gene.cor.pvalue[upper.tri(gene.cor.pvalue)] = NA

gene.cor.cor.melt <- melt(gene.cor.cor) #gene1 \t gene2 \t cor

gene.cor.pvalue.melt <- melt(gene.cor.pvalue)

gene.melt <- data.frame(from = gene.cor.cor.melt$Var2,to=gene.cor.cor.melt$Var1,cor=gene.cor.cor.melt$value,pvalue=gene.cor.pvalue.melt$value)

gene.melt <- gene.melt[gene.melt$from!=gene.melt$to&!is.na(gene.melt$pvalue),,drop=F]

gene.edge <- gene.melt[gene.melt$pvalue<0.0001,,drop=F]

gene.edge$color <- ifelse(gene.edge$cor>0,'pink','#6495ED')

gene.edge$weight <- abs(gene.edge$cor)*6

gene.node <- gene.group

group.color <- colorRampPalette(brewer.pal(9, "Set1"))(length(unique(gene.node$group)))

gene.node$color <- group.color[as.numeric(as.factor(gene.node$group))]

gene.node$shape <- "circle"

gene.node$frame <- ifelse(gene.cox$HR>1,'purple',"green")

gene.node$pvalue <- gene.cox$pvalue

pvalue.breaks <- c(0,0.0001,0.001,0.01,0.05,1)

pvalue.size <- c(16,14,12,10,8)

cutpvalue <- cut(gene.node$pvalue,breaks=pvalue.breaks)

gene.node$size <- pvalue.size[as.numeric(cutpvalue)]

nodefile <- "network.node.txt"

edgefile <- "network.edge.txt"

write.table(gene.node, nodefile, sep="\t", col.names=T, row.names=F, quote=F)

write.table(gene.edge, edgefile, sep="\t", col.names=T, row.names=F, quote=F)

node = read.table(nodefile, header=T, sep="\t", comment.char="")

edge = read.table(edgefile, header=T, sep="\t", comment.char="")

g = graph.data.frame(edge,directed = FALSE)

node = node[match(names(components(g)$membership),node$id),]

if(!is.na(match('color',colnames(node)))) V(g)$color = node$color

if(!is.na(match('size',colnames(node)))) V(g)$size = node$size

if(!is.na(match('shape',colnames(node)))) V(g)$shape = node$shape

if(!is.na(match('frame',colnames(node)))) V(g)$frame = node$frame

pdf(file="network.pdf", width=10, height=8)

par(mar=c(0,0,0,0))

layout(matrix(c(1,1,4,2,3,4),nc=2),height=c(4,4,2),width=c(8,3))

coord = layout_in_circle(g)

degree.x = acos(coord[,1])

degree.y = asin(coord[,2])

degree.alpha = c()

for(i in 1:length(degree.x)){

if(degree.y[i]<0) degree.alpha=c(degree.alpha,2*pi-degree.x[i]) else degree.alpha=c(degree.alpha,degree.x[i])

}

degree.cut.group = (0:8)/4*pi

degree.cut.group[1] = -0.0001

degree.cut = cut(degree.alpha,degree.cut.group)

degree.degree = c(-pi/4,-pi/4,-pi/2,-pi/2,pi/2,pi/2,pi/2,pi/4)

degree = degree.degree[as.numeric(degree.cut)]

values <- lapply(node$id,function(x)c(1,1))

V(g)$pie.color = lapply(1:nrow(node),function(x)c(node$color[x],node$frame[x]))

V(g)$frame = NA

plot(g,layout=layout_in_circle,vertex.shape="pie",vertex.pie=values,

vertex.label.cex=V(g)$lable.cex,edge.width = E(g)$weight,edge.arrow.size=0,

vertex.label.color=V(g)$color,vertex.frame.color=V(g)$frame,edge.color=E(g)$color,

vertex.label.cex=2,vertex.label.font=2,vertex.size=V(g)$size,edge.curved=0.4,

vertex.color=V(g)$color,vertex.label.dist=1,vertex.label.degree=degree)

The default value is -pi/4

par(mar=c(0,0,0,0))

plot(1,type="n",xlab="",ylab="",axes=F)

groupinfo = unique(data.frame(group=node$group,color=node$color))

legend("left",legend=groupinfo$group,col=groupinfo$color,pch=16,bty="n",cex=3)

par(mar=c(0,0,0,0))

plot(1,type="n",xlab="",ylab="",axes=F)

legend("left",legend=c('Risk factors','Favorable factors'),col=c('purple','green'),pch=16,bty="n",cex=2.5)

par(mar=c(0,0,0,0))

plot(1,type="n",xlab="",axes=F,ylab="")

legend("top",legend=c('Postive correlation with P<0.0001','Negative correlation with P<0.0001'),lty=1,lwd=4,col=c('pink','#6495ED'),bty="n",cex=2.2)

legend('bottom',legend=c(0.0001,0.001,0.01,0.05,1),pch=16,pt.cex=c(1.6,1.4,1.2,1,0.8)*6,bty="n",ncol=5,cex=2.2,col="black",title="Cox test, pvalue")

dev.off()

**Figure 5 Related code is as follows:**

library(limma)

library(ggpubr)

ICDCluFile="7.txt"

geneCluFile="14.txt"

scoreFile="19.txt"

ICDClu=read.table(ICDCluFile, header=T, sep="\t", check.names=F, row.names=1)

geneClu=read.table(geneCluFile, header=T, sep="\t", check.names=F, row.names=1)

score=read.table(scoreFile, header=T, sep="\t", check.names=F, row.names=1)

twoCluster=cbind(ICDClu, geneClu)

rownames(twoCluster)=gsub("(.*?)\\_(.*?)", "\\2", rownames(twoCluster))

sameSample=intersect(row.names(twoCluster), row.names(score))

data=cbind(score[sameSample,,drop=F], twoCluster[sameSample,,drop=F])

data$ICDcluster=factor(data$ICDcluster, levels=levels(factor(data$ICDcluster)))

group=levels(factor(data$ICDcluster))

comp=combn(group, 2)

my_comparisons=list()

for(i in 1:ncol(comp)){my_comparisons[[i]]<-comp[,i]}

bioCol=c("#0066FF","#FF9900","#FF0000","#6E568C","#7CC767","#223D6C","#D20A13","#FFD121","#088247","#11AA4D")

bioCol=bioCol[1:length(levels(factor(data$ICDcluster)))]

boxplot=ggboxplot(data, x="ICDcluster", y="riskScore", color="ICDcluster",

xlab="ICDcluster",

ylab="Risk score",

legend.title="ICDcluster",

palette=bioCol,

add = "jitter")+

stat_compare_means(comparisons = my_comparisons)

pdf(file="Figure5A.pdf.pdf", width=5, height=4.5)

print(boxplot)

dev.off()

data$geneCluster=factor(data$geneCluster, levels=levels(factor(data$geneCluster)))

group=levels(factor(data$geneCluster))

comp=combn(group, 2)

my_comparisons=list()

for(i in 1:ncol(comp)){my_comparisons[[i]]<-comp[,i]}

bioCol=c("#0066FF","#FF9900","#FF0000","#6E568C","#7CC767","#223D6C","#D20A13","#FFD121","#088247","#11AA4D")

bioCol=bioCol[1:length(levels(factor(data$geneCluster)))]

boxplot=ggboxplot(data, x="geneCluster", y="riskScore", color="geneCluster",

xlab="geneCluster",

ylab="Risk score",

legend.title="geneCluster",

palette=bioCol,

add = "jitter")+

stat_compare_means(comparisons = my_comparisons)

pdf(file="Figure5B.pdf", width=5, height=4.5)

print(boxplot)

dev.off()

#???ð?

library(ggalluvial)

library(ggplot2)

library(dplyr)

ICDCluFile="7.txt"

geneCluFile="14.txt"

riskFile="19.txt"

ICDClu=read.table(ICDCluFile, header=T, sep="\t", check.names=F, row.names=1)

geneClu=read.table(geneCluFile, header=T, sep="\t", check.names=F, row.names=1)

risk=read.table(riskFile, header=T, sep="\t", check.names=F, row.names=1)

twoCluster=cbind(ICDClu, geneClu)

rownames(twoCluster)=gsub("(.*?)\\_(.*?)", "\\2", rownames(twoCluster))

sameSample=intersect(row.names(twoCluster), row.names(risk))

rt=cbind(risk[sameSample,,drop=F], twoCluster[sameSample,,drop=F])

rt=rt[,c("ICDcluster", "geneCluster", "risk", "fustat")]

colnames(rt)=c("ICDcluster", "geneCluster", "Risk", "Fustat")

rt[,"Fustat"]=ifelse(rt[,"Fustat"]==0, "Alive", "Dead")

corLodes=to_lodes_form(rt, axes = 1:ncol(rt), id = "Cohort")

pdf(file="Figure5C.pdf", width=6, height=5.5)

mycol=rep(c("#0066FF","#FF9900","#FF0000","#029149","#6E568C","#E0367A","#D8D155","#223D6C","#D20A13","#431A3D","#91612D","#FFD121","#088247","#11AA4D","#58CDD9","#7A142C","#5D90BA","#64495D","#7CC767"),15)

ggplot(corLodes, aes(x = x, stratum = stratum, alluvium = Cohort,fill = stratum, label = stratum)) +

scale_x_discrete(expand = c(0, 0)) +

#??aes.flow??????????ɫ??forward˵????ɫ??ǰ??????״ͼһ?£?backward˵???ͺ???????״ͼһ?¡?

geom_flow(width = 2/10,aes.flow = "forward") +

geom_stratum(alpha = .9,width = 2/10) +

scale_fill_manual(values = mycol) +

#size=3??????????С

geom_text(stat = "stratum", size = 3,color="black") +

xlab("") + ylab("") + theme_bw() +

theme(axis.line = element_blank(),axis.ticks = element_blank(),axis.text.y = element_blank()) + #ȥ????????

theme(panel.grid =element_blank()) +

theme(panel.border = element_blank()) +

ggtitle("") + guides(fill = FALSE)

dev.off()

library(reshape2)

library(ggpubr)

riskFile="19.txt" #?????ļ?

estimateFile="TMEscores.txt" #????΢?????????ļ?

Risk=read.table(riskFile, header=T, sep="\t", check.names=F, row.names=1)

Risk$risk=factor(Risk$risk, levels=c("low","high"))

score=read.table(estimateFile, header=T, sep="\t", check.names=F, row.names=1)

score=score[,1:3]

rownames(score)=gsub("(.*?)\\_(.*?)", "\\2", rownames(score))

score=score[row.names(Risk),,drop=F]

rt=cbind(Risk[,"risk",drop=F], score)

data=melt(rt, id.vars=c("risk"))

colnames(data)=c("Risk", "scoreType", "Score")

p=ggviolin(data, x="scoreType", y="Score", fill = "Risk",

xlab="",

ylab="TME score",

legend.title="Risk",

add = "boxplot", add.params = list(color="white"),

palette = c("blue","red"), width=1)

p=p+rotate_x_text(45)

p1=p+stat_compare_means(aes(group=Risk),

method="wilcox.test",

symnum.args=list(cutpoints = c(0, 0.001, 0.01, 0.05, 1), symbols = c("***", "**", "*", " ")),

label = "p.signif")

pdf(file="Figure5D.pdf", width=6, height=5)

print(p1)

dev.off()

library(ggpubr)

library(reshape2)

tmbFile="21.txt"

riskFile="19.txt"

cluFile="14.txt"

tmb=read.table(tmbFile, header=T, sep="\t", check.names=F, row.names=1)

risk=read.table(riskFile, header=T, sep="\t", check.names=F, row.names=1)

clu=read.table(cluFile, header=T, sep="\t", check.names=F, row.names=1)

clu=read.table(cluFile, header=T, sep="\t", check.names=F, row.names=1)

tmb=as.matrix(tmb)

tmb[tmb>quantile(tmb,0.975)]=quantile(tmb,0.975)

sameSample=intersect(row.names(tmb), row.names(risk))

tmb=tmb[sameSample,,drop=F]

risk=risk[sameSample,,drop=F]

rownames(clu)=gsub("(.*?)\\_(.*?)", "\\2", rownames(clu))

clu=clu[sameSample,,drop=F]

data=cbind(risk, tmb, clu)

data=data[,c("riskScore", "risk", "geneCluster", "TMB")]

data$risk=factor(data$risk, levels=c("low", "high"))

risk=levels(factor(data$risk))

comp=combn(risk, 2)

my_comparisons=list()

for(i in 1:ncol(comp)){my_comparisons[[i]]<-comp[,i]}

bioCol=c("#0066FF","#FF0000","#6E568C","#7CC767","#223D6C","#D20A13","#FFD121","#088247","#11AA4D")

bioCol=bioCol[1:length(risk)]

boxplot=ggboxplot(data, x="risk", y="TMB", fill="risk",

xlab="",

ylab="Tumor Burden Mutation",

legend.title="Risk",

palette = bioCol )+

stat_compare_means(comparisons = my_comparisons)

pdf(file="Figure5E.pdf",width=5,height=4.5)

print(boxplot)

dev.off()

length=length(levels(factor(data$geneCluster)))

bioCol=c("#0066FF","#FF9900","#FF0000","#6E568C","#7CC767","#223D6C","#D20A13","#FFD121","#088247","#11AA4D")

p1=ggplot(data, aes(riskScore, TMB)) +

xlab("Risk score")+ylab("Tumor Burden Mutation")+

geom_point(aes(colour=geneCluster))+

scale_color_manual(values=bioCol[1:length])+

geom_smooth(method="lm",formula = y ~ x) + theme_bw()+

stat_cor(method = 'spearman', aes(x =riskScore, y =TMB))

pdf(file="Figure5F.pdf", width=6, height=4.5)

print(p1)

dev.off()

library(maftools)

risk=read.table("19.txt", header=T, sep="\t", check.names=F)

outTab=risk[,c(1, ncol(risk))]

colnames(outTab)=c("Tumor_Sample_Barcode", "Risk")

write.table(outTab, file="ann.txt", sep="\t", quote=F, row.names=F)

geneNum=20

geneMut=read.table("geneMut.txt", header=T, sep="\t", check.names=F, row.names=1)

gene=row.names(geneMut)[1:geneNum]

ann_colors=list()

col=c("blue", "red")

names(col)=c("low", "high")

ann_colors[["Risk"]]=col

pdf(file="Figure5M.pdf", width=6, height=6)

maf=read.maf(maf="low.maf", clinicalData="ann.txt")

oncoplot(maf=maf, clinicalFeatures="Risk", genes=gene, annotationColor=ann_colors, keepGeneOrder=T)

dev.off()

pdf(file="Figure5N.pdf", width=6, height=6)

maf=read.maf(maf="high.maf", clinicalData="ann.txt")

oncoplot(maf=maf, clinicalFeatures="Risk", genes=gene, annotationColor=ann_colors, keepGeneOrder=T)

dev.off()

#???ð?

library(limma)

library(ggplot2)

library(ggpubr)

library(ggExtra)

riskFile="19.txt"

RNAssFile="StemnessScores_RNAexp_20170127.2.tsv" #??ϸ???????ļ?

risk=read.table(riskFile, header=T, sep="\t", check.names=F, row.names=1)

RNAss=read.table(RNAssFile, header=T, sep="\t",check.names=F, row.names=1)

RNAss=t(RNAss[1,,drop=F])

rownames(RNAss)=gsub("(.*?)\\-(.*?)\\-(.*?)\\-.*", "\\1\\-\\2\\-\\3", rownames(RNAss))

RNAss=avereps(RNAss)

sameSample=intersect(row.names(risk), row.names(RNAss))

risk=risk[sameSample,"riskScore",drop=F]

RNAss=RNAss[sameSample,,drop=F]

data=cbind(RNAss, risk)

xlab="riskScore"

ylab="RNAss"

outFile="Figure5O.pdf"

x=as.numeric(data[,xlab])

x[x>quantile(x,0.99)]=quantile(x,0.99)

y=as.numeric(data[,ylab])

df1=as.data.frame(cbind(x,y))

p1=ggplot(df1, aes(x, y)) +

xlab("Risk score") + ylab(ylab)+ ylim(0,0.7)+

geom_point() + geom_smooth(method="lm",formula = y ~ x) + theme_bw()+

stat_cor(method = 'spearman', aes(x =x, y =y))

p2=ggMarginal(p1, type="density", xparams=list(fill = "orange"), yparams=list(fill = "blue"))

pdf(file=outFile, width=5.2, height=5)

print(p2)

dev.off()

Figure 6 as part of machine learning, in the relevant code and file can be used by *https://github.com/uro66/postgraduate* query.
