# Supplementary material for: Subtype and prognostic analysis of immunogenic cell death-related gene signature in prostate cancer
Source: Front Oncol. 2023 Jun 6;13:1160972. doi: 10.3389/fonc.2023.1160972 (PMC10279955; doi:10.3389/fonc.2023.1160972)
Supplement: Supplementary file 2 [file DataSheet_2.pdf]

A total of two sheets of gels (Figure 1) were used for this western blot experiment, with three markers incubated on the left side of each gel, one marker incubated on the right side, and C4-2 and RPEW-1 proteins incubated in the middle. After comparing with the internal control GAPDH and determining that there was no significant difference in the internal control, the membrane was reblocked after stripping the antibody and the CST2 and SERPINA3 antibodies were incubated, respectively(Figure 2). The protein expression in different prostate tissues were observed.

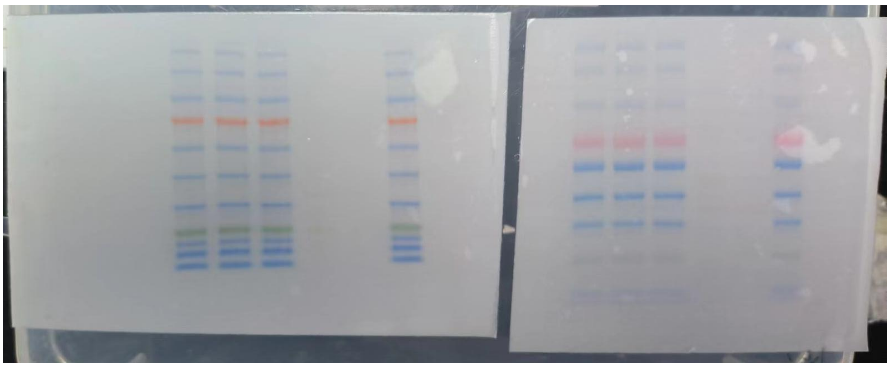

Figure 1 gels

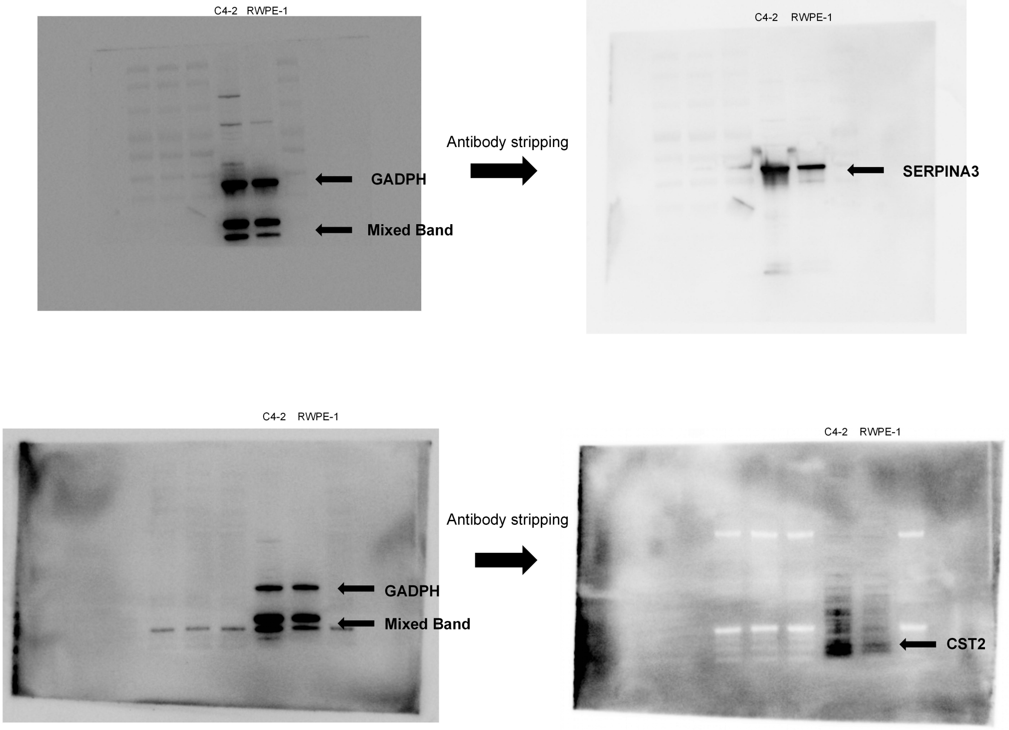

Figure 2 Antibody stripping procedure
